# Behaviour change interventions to reduce opioids in chronic non-cancer pain in primary care: A systematic review and mapping of behavior change techniques

**DOI:** 10.1097/MD.0000000000050106

**Published:** 2026-08-07

**Authors:** Andreia Ramos Silva, Catharine Montgomery, Bernhard Frank, Katie Herron, Helen Poole

**Affiliations:** a School of Psychology, Faculty of Health, Liverpool John Moores University, Liverpool, United Kingdom; b Department of Pain Medicine, The Walton Centre NHS Foundation Trust, Liverpool, United Kingdom; c Pain Management Programme, The Walton Centre NHS Foundation Trust, Liverpool, United Kingdom.

**Keywords:** chronic pain, opioid, primary care, psychosocial interventions

## Abstract

**Background:**

Despite limited efficacy, patients are prescribed opioids for chronic non-cancer pain (CNCP). Guidelines recommend supporting the safe tapering of opioid prescriptions for these patients. This usually occurs in primary care, so there is a need to ascertain the efficacy of interventions implemented in this setting. This review aims to evaluate the effectiveness of interventions to reduce opioids in CNCP patients in primary care and identify effective behavior change techniques (BCTs).

**Methods:**

A comprehensive search was conducted in Medline, Cumulative Index to Nursing and Allied Health Literature and Web of Science databases in May 2023 using the MESH terms: “Chronic pain,” “opioid,” “primary care,” and “psychosocial interventions.” Manual searches were conducted in 7 systematic reviews. Psychosocial intervention trials with a control group were eligible for the review. Studies were excluded if opioid dosages were not reported.

**Results:**

Thirteen studies were included - 7 randomized controlled trials and 6 cohort studies with a total of 3130 patients. Upon quality assessment, one study was deemed strong, two were moderate, and ten were of weak quality. Eight interventions demonstrated success in opioid tapering in primary care settings. A broad spectrum of intervention techniques were identified, with *Behaviour Substitution* consistently applied in the effective interventions.

**Conclusion:**

We determined the effective BCTs used across these trials to guide design and implementation of future interventions. This is the first review to identify BCTs that may contribute to supporting patients in tapering opioids in CNCP in primary care settings. Limitations include high heterogeneity in study designs and in the reporting of techniques used.

## 1. Introduction

Chronic non-cancer pain (CNCP) has been defined as pain that lasts longer than 3 months and continues after the considered normal time of tissue healing. People affected by chronic pain experience pain on most days, or every day, which impairs their daily activities.^[1]^ Also, chronic pain is in the top ten leading causes of disease burden and disability worldwide by the Global Burden of Disease Reviews, including low back pain, headache and neck pain.^[2,3]^ Worldwide, it is estimated that 30% of the population suffers from chronic pain, and that explains up to 20% of healthcare visits.^[1,4]^ In the United Kingdom (UK), it is estimated that 43% of people are affected by chronic pain and in about 15% of people, the pain is considered to be severe and disabling, causing significant impairment in their daily activities.^[5]^

Despite the limited evidence that opioids are effective in the treatment of chronic pain,^[6–8]^ opioid medication has been the first line of treatment for chronic pain for many years.^[9,10]^ A recent systematic review identified that 31% of the patients with CNCP are prescribed opioids, with strong opioids being prescribed 18% more frequently than weak opioids 8.5%.^[11]^ Epidemiological studies conducted in the UK, United States (US), Canada and Australia have raised awareness of the increase in opioid prescriptions in the last decades^[12–16]^ alongside misuse of prescribed opioids and the significant rise in related deaths.^[17]^

Furthermore, long-term opioid treatment has been associated with serious harms such as falls and fractures, road accidents, sleep apnea, hyperalgesia, immunosuppression, chronic constipation, higher risk of myocardial infarction and erectile dysfunction.^[18,19]^ Likewise, the evidence synthesis from the Centers for Disease Control in the US showed that opioid prescriptions were associated with addiction, overdose and death.^[7,8]^

Despite the growing evidence that long-term opioids are not effective for CNCP and can increase the risk of harm, they continue to be prescribed, especially in primary care where most CNCP patients are seen. In the UK, from 2000 to 2010 there was a 466% rise in opioid prescriptions in primary care, with 88% of the strong opioids being prescribed for CNCP and only 12% were for cancer-related pain. Morphine was the most prescribed, but with a marked increase also in the prescription of oxycodone, buprenorphine and fentanyl.^[10]^ Another study in the UK^[20]^ shows that between 2005 and 2012 in primary care, prescription of weak opioids doubled while prescriptions of strong opioids have risen sixfold during this time frame. Furthermore, from 2006 to 2017 opioid prescriptions increased 5-fold, with codeine, dihydrocodeine and tramadol being the mainly prescribed opioids. In addition, oxycodone prescriptions increased 30-fold over 12 years in one study in primary care centers around the UK.^[21]^ However, the same study reports that rates for prescription of dihydrocodeine, tramadol and fentanyl were steady until 2017. Similarly, another study analyzing UK primary care prescription data reported a 34% increase in opioid prescriptions with a 127% increase in morphine equivalent dose (MED) between 1998 and 2016 and a decrease in prescriptions in the years 2016 to 2017.^[22]^ This decrease in the prescription of opioids coincides with the publication of guidelines in the US by the Centers for Disease Control to limit opioid prescribing for CNCP.^[7,8]^

Similarly, in the UK, the National Institute for Health and Care Excellence guidelines^[23]^ published recommendations in favor of person-centered non-pharmacological treatment of CNCP and recommended safe reduction or stopping opioids in patients that opioids no longer benefit or are at risk of harm. However, these guidelines do not recommend any particular intervention to support these patients and guidance on how to support these patients in primary care is lacking.^[23,24]^ Reducing opioids for CNCP is complex due to various biological, psychological, and social factors implicated in the pain experience.^[25–28]^ Psychosocial interventions have shown potential in supporting various health problems, including pain management. Furthermore, for chronic pain they can support patients to modify maladaptive behaviors and adopt more helpful thought patterns and emotional responses to pain.^[29–34]^ These interventions target psychological and behavioral factors, including emotional regulation, self-efficacy and pain-related distress, and equip patients with coping and pain management skills which can support pain management and opioid medication use.^[35–39]^ Such interventions incorporate behavior change techniques (BCTs), which are specific strategies that aid in modifying the processes that drive behavior.^[40,41]^

Although these techniques are complex and difficult to isolate, by clearly identifying and categorizing them, we can understand which strategies have been successful in reducing opioids and improving pain management. To this end, Michie and coworkers^[41,42]^ introduced a taxonomy to categorize BCTs. Moreover, it is essential to recognize these techniques to promote an evidence-based approach to inform the development of future interventions for reducing opioid reliance in CNCP. Interventions designed with BCTs as a foundation can offer structured support to enhance patient self-management and reduce opioid dependence. Since most CNCP patients are managed in Primary Care rather than specialist pain services, integrating such design within primary care settings is essential.^[21,43,44]^ This can alleviate pressure on the healthcare system by reducing reliance on General Practitioners (general practitioners - family doctors; primary care physicians) for medication management and minimizing unnecessary referrals to specialist services, ensuring more efficient care. Specialist services provide intensive interventions to patients with more complex pain presentations and addictions.^[45]^ Furthermore, long waiting times limit access to specialist care for many patients who might benefit from opioid reduction.^[46,47]^

Therefore, this review maps techniques used in psychosocial interventions to the BCT taxonomy to contribute knowledge to the design of future interventions. Despite former systematic reviews investigating the effectiveness of interventions in reducing opioids in CNCP, none of them focused on behavior strategies applied to reduce opioids in CNCP in primary care. Additionally, this review adds more recent literature (after January 2021) to the most recently published reviews. This review aims to evaluate and synthesize current evidence by examining the effectiveness of psychosocial interventions and BCTs for CNCP in reducing opioids in primary care.

## 2. Methods

This report consists of a systematic review and narrative synthesis. Since previous systematic reviews of interventions to reduce opioids have been published in recent years, the starting point of this review was a review of Systematic Reviews^[24,36,48–52]^ followed by database searches.

### 2.1. Inclusion criteria for the studies

- Studies reporting a psychosocial or behavioral intervention for chronic pain with support to reduce/stop opioids in CNCP;
- Randomised controlled trials, quasi-experimental studies and preexperimental studies (that meet the other inclusion criteria);
- Participants: adults with CNCP;
- At least one BCT;
- Studies conducted in primary care (including general practitioner services or community services)
- Adults (18 years old or over) participants;
- Studies with the aim or outcome to reduce opioids and/or reports of opioid tapering doses are reported.

### 2.2. Exclusion

- Does not report opioid tapering/withdrawal doses at baseline and end of treatment or follow-up;
- Studies conducted in secondary care such as hospitals and pain centers;
- Studies conducted in long-term care or institutional care;
- Studies published more than 10 years ago (before 2011).

### 2.3. Study design plan

This review adheres to Preferred Reporting Items for Systematic Reviews and Meta-Analyses (PRISMA) guidelines using the Population, Intervention, Comparison, Outcomes, Study Designs, and Timeframe components.

#### 2.3.1. Population

Adults (aged over 18 years) with CNCP who were prescribed opioids. Including all types of chronic nonmalignant pain.

Studies that took place in primary care or community healthcare settings.

#### 2.3.2. Interventions

Studies that focused on the management of pain and/or management of withdrawal of prescribed opioids. Opioid withdrawal was defined as the planned and supervised process of gradually reducing or stopping the opioids that no longer sustain advantages for the patient. Studies that the first outcome was not opioid withdrawal but did report opioid dosages at different time points were included.

#### 2.3.3. Comparators

Studies that compared a psychosocial intervention to usual care, other interventions, or those that did not use a comparator were considered for inclusion.

#### 2.3.4. Outcomes

The studies included in the analysis reported the dosage of opioids at the beginning and end of the intervention or follow-up period.

#### 2.3.5. Study designs

All types of studies that feature a psychosocial intervention with behavioral strategies and reported on opioid dosage were eligible for inclusion. Study types considered for inclusion were randomized controlled trials, quasi-experimental, and observational studies.

#### 2.3.6. Timeframe

Studies published since 2011 were included. Older interventions may not have the same applicability in the current systems.

### 2.4. Data source and search strategy

Seven Systematic Reviews on reducing opioids in CNCP were identified, and the studies included in each systematic review were screened, removing duplicates and studies published before 2011. Database searches were conducted on Medline (Ebsco), Cumulative Index to Nursing and Allied Health Literature (Ebsco), Scopus and Web of Science. “Chronic Pain,” “Opioid,” “Psychosocial Intervention” and ‘Primary Care’ were the main terms used for controlled vocabulary searches. The search strategy was adapted to the different databases and reviewed by the University research librarian. Database searches were conducted on the 30^th^ and 31^st^ of May 2023 and included records since January 2021 as per the last date that searches were conducted on the analyzed systematic reviews.^[48]^ Records were filtered for only adults, only the English language and published since January 2021 (File S1, Supplemental Digital Content 1 - Search Strategy).

### 2.5. Data extraction

The studies were screened using abstracts, and then full text, and studies meeting the inclusion criteria were retained. Where uncertainty arose on the inclusion of specific studies, HP and CM reviewed the studies independently and discussed them until a consensus was reached.

Study characteristics were extracted from each study and collated in a Microsoft Excel spreadsheet. The following data elements were extracted: Title, author, country of delivery, year of publication, details of the intervention, mode of delivery group or individual, population, mean age, mean sex, intervention, comparator, opioid dosage at baseline, opioid dosage at end of treatment, primary and secondary outcomes, study design, setting, opioid measure, measures, duration/frequency, follow-up and findings/results.

When there were several studies from the same trial, this information was merged in order to obtain the full picture of the intervention. Where additional information was required, the corresponding authors were contacted by email. Authors were first contacted during study selection to clarify eligibility. If eligible, authors were then contacted following inclusion of the final studies to obtain additional information to support BCT coding.

### 2.6. Quality assessment

Quality assessment of all included studies was conducted using the Effective Public Health Practice Project, which is advised for use in Public Health interventions.^[53]^

The tool helps to assess selection bias, study design, confounding variables, blinding, data collection methods, withdrawals and dropout reasons and final overall grade. The domains are graded strong, moderate or weak based on the details reported in the study. The final quality rating is then determined as Strong (no weak ratings), Moderate (one weak rating) or Weak (two or more weak ratings).

### 2.7. Data mapping to behavioural change techniques taxonomy

In this review, we have categorized each intervention’s BCTs according to the BCTs-Taxonomy v1 using the 93 techniques framework.^[41,42]^ This task was accomplished by identifying the BCTs and linking them within the 93 categories. This process is interpretative of the description of the techniques used by the authors, and it requires a qualitative classification of the information presented. To ensure accuracy in the process, the author (ARS) completed training in identifying and coding BCTs (bct.taxonomy.com).

If a study presented information related to a specific technique from the taxonomy, this was cross-referenced with the list and marked as instructed in the BCT taxonomy training. It is recommended to mark a technique with “X+” when not certain if it has been used and “X++” if confident that the technique has been applied. This task has been carried out using the Taxonomy mobile app for assistance.

The corresponding authors of the thirteen eligible studies included in the SR were contacted requesting additional information to accurately map the techniques to the taxonomy. 6 authors replied with further information, such as supplementary materials or a more detailed explanation of the intervention components. Where no response was received, BCT coding was based on the published article and any available supplementary materials. The supplementary materials and the studies were reread until the authors were confident in the mapping process.

## 3. Results

### 3.1. Types of studies

In total, 219 studies were retrieved across the systematic reviews, 61 duplicates were removed, and 80 studies were excluded by year of publication. A total of 7 studies extracted from the systematic reviews were kept for this review. Databases were searched from January 2021 to the 31^st^ May 2023. Database searches retrieved 145 studies; 40 duplicates were removed, and six fulfilled the inclusion criteria for this review (see PRISMA flow diagram and supplementary PRISMA checklist).

### 3.2. PRISMA flow diagram

All abstracts were screened, and if they potentially met the inclusion criteria, the text was read in full. Ten corresponding authors were emailed requesting more information on the inclusion criteria, particularly opioid dosage outcomes or intervention type, which were unclear (.^[54–63]^ Five authors replied with additional information relating to inclusion criteria. Following review of the information provided, two of these studies^[54,56]^ met the inclusion criteria because the required opioid dosage data were provided. Upon review, a total of thirteen studies were included in this analysis from the database searches and systematic review. The designs included randomized controlled trials (RCTs), single-arm studies and prospective cohort studies.

### 3.3. Study characteristics

The studies involved a total of n = 3130 patients, with individual samples ranging from n = 34 to n = 850. Out of the 13 studies, eight are RCTs, two are quality improvement studies with matched prospective cohorts, and three are single-arm prospective cohort studies. Six new RCTs were not included in previous systematic reviews (see Study Characteristics in Table S1, Supplemental Digital Content 2).

Of the thirteen studies, eleven were conducted in the US, and two in the UK.^[64–66]^ All studies were delivered in primary care centers, community centers or primary care centers for veterans. Three interventions were conducted with veterans in veteran primary care centers.^[54,56,67]^ From the 13 studies, 6 interventions were delivered individually to patients^[54,56,67–70]^ five in a group^[71–75]^ and two in a mix of group and individual interventions.^[65,66]^ The delivery mode of the interventions was diverse, as it ranged from 7 interventions in-person,^[66,68,71–75]^ 4 mixes of in-person and telephone delivery,^[54,56,65,69]^ one in-person and telehealth (video call)^[67]^ and one web-based (videos).^[70]^ The person delivering the intervention varied from primary care doctors,^[68]^ to multidisciplinary teams,^[56,67,73–75]^ project workers,^[66]^ psychiatry pain doctor, doctor and doctor assistant^[69]^; researchers clinical workers^[71,72]^; trained nurses^[54]^; trained nurses and layperson with CNCP and experience in tapering opioids^[65]^ and online videos (psychologists).^[70]^

MED at baseline ranged from a median of 17.5mg interquartile range (IQR) (0–120mg) (Vogler et al 2017) up to 288 mg (IQR (153–288)^[68]^ (see Opioid Dosage outcome in Table S2, Supplemental Digital Content 3). Of the 13 studies, eight had a decrease in the opioid dose as the main outcome, three studies had opioid reduction as a secondary outcome and 2 studies as an exploratory outcome. Other primary outcomes of the interventions focused on opioid misuse risk, pain interference, pain intensity, self-reported pain impact, health and wellbeing and quality of life (QoL), healthcare services usage, and functional status (Table S1, Supplemental Digital Content 2). The studies had different approaches to opioid dose reduction; 4 interventions included tapering plans in their protocol, in 2 interventions opioid tapering was suggested to patients, but a tapering plan was not devised, and 7 interventions did not have a tapering protocol, and it was left to the patients’ decision to wean off opioids (Table S2, Supplemental Digital Content 3).

In twelve studies, patients had a variety of CNCP, and in one study only patients with chronic low back pain were recruited.^[54]^ The comparison groups were control groups such as usual care (n = 3), Attention Control with Psychoeducation,^[56]^ enhanced usual care,^[65]^ cognitive behavioral therapy (CBT),^[54]^ Active Support Group,^[72]^ Supportive Psychotherapy Group,^[71]^ matched cohort (n = 2) and no comparator (n = 3) (see Table S1, Supplemental Digital Content 2).

### 3.4. Study quality

Table 1 below shows the Study Quality Assessment with the Effective Public Health Practice Project checklist criteria. The majority of studies reviewed were assessed as being of weak quality. See File S2, Supplemental Digital Content 4 for a detailed Quality Assessment of each study.

**Table 1:** Study quality assessment.

Studies	Selection bias	Study design	Confounders	Blinding	Data collection methods	Withdrawal and dropouts	Total score
Borsari et al 2021	Weak	Strong	Moderate	Moderate	Strong	Strong	Moderate
Bushey et al 2022	Moderate	Strong	Weak	Moderate	Strong	Strong	Moderate
Darnall et al 2018	Weak	Moderate	N/A	Weak	Weak	Moderate	Weak
DeBar et al 2022	Weak	Strong	Strong	Moderate	Strong	Weak	Weak
Garland et al 2020	Weak	Strong	Strong	Moderate	Strong	Weak	Weak
Garland et al 2022	Moderate	Strong	Strong	Moderate	Strong	Strong	Strong
Mehl-Madrona et al 2016	Moderate	Moderate	Strong	Weak	Strong	Weak	Weak
Sandhu et al 2023	Weak	Strong	Strong	Weak	Strong	Weak	Weak
Scott et al 2020	Weak	Moderate	^a^N/A	Weak	Strong	Weak	Weak
Seal et al 2020	Weak	Moderate	Strong	N/A	Strong	N/A	Moderate
Sullivan et al 2017	Weak	Strong	Strong	Weak	Strong	Strong	Weak
Vogler et al 2017	Weak	Moderate	Weak	Weak	Weak	Weak	Weak
Wilson et al 2023	Moderate	Strong	Strong	Weak	Strong	Strong	Moderate

N/A- Not applicable.

### 3.5. Opioid dosage outcome

The studies included in this review used converted measures of opioid dosage to MED Dose. Most of the studies used medical records to assess the opioid prescription dosage at baseline and end of treatment (see Table S2, Supplemental Digital Content 3). Generally, the studies reported mixed results in reducing opioid prescription. Six studies reported statistically significant reductions after the intervention between groups^[65,67,70–72,74]^ and three reported significant reductions pre- and post-intervention ^[66,68,74]^ see Table S2, Supplemental Digital Content 3.

### 3.6. Opioid misuse risk

Five studies included in this review measured Opioid Misuse Risk.^[54,56,70–72]^ All of the studies used the Current Opioid Misuse Measure Scale except Garland^[71]^ which used a Drug Misuse Index that included the Current Opioid Misuse Measure and Addiction Behaviours Checklist and Urine toxicology test. Out of the 5 intervention studies, only the 2 studies from Garland^[71,72,76]^ showed statistically significant changes in opioid misuse risk. The studies reported improvements from baseline to follow-up at 3 months^[72,76]^ and at 9 months^[71]^ in the intervention group in relation to the comparator group. Also, the intervention group^[71]^ showed a higher proportion of participants (45%) effectively stopping opioid misuse (see Table 2 below).

**Table 2:** Opioid misuse outcomes in the intervention studies with differences between intervention groups.

Study	Outcome measure	Mean differences (95% CI) between groups	*P*-value
**Borsari et al. 2021**	^a^COMM	No differences, NA	Not sig, NA
**Bushey et al (2022**)	COMM	−1.37 (-3.38 to 0.65)	.12
**Garland et al (2019**)	COMM	−3.03 (−5.71, −0.34)	.027
**Garland et al (2022**)	DMI	OR: 2.06 (1.17–3.61) Risk diff: 0.15	.01
**Wilson et al (2023**)	COMM	0.00 (−0.20,0.21)	.94

COMM = current opioid misuse measure, DMI = drug misuse index (COMM, Addiction Behaviours Checklist and Urine toxicology test), NA = not available, not sig- not significant, CI = confidence interval, OR = odds ratio.

### 3.7. Pain impact

Ten of the included studies measured Pain Impact and used different measures, including the Brief Pain Inventory, Patient-Reported Outcomes Measurement Information System, Pain, Enjoyment of Life and General Activity Scale, and other visual pain scales, with the results of these summarized in Table 3 below. Six studies showed significant improvements in Pain Impact (see Table 3). Two trials showed improvements at the. Another trial study noted a significant reduction within the intervention group in pain impact from baseline to after the intervention (22 weeks), but this was not maintained at follow-up (34 weeks), and there were no differences between groups. Three studies did not include follow-up.

**Table 3:** Pain impact outcomes in the intervention studies with differences between intervention groups and control groups.

Study	Outcome measure	Mean differences (95% CI) between groups	*P*-value
**Borsari et al (2021**)	Pain severity (BPI)	d = 0.33 (CCMI) d = 0.36 (ACP)	NA
	Pain interference (BPI)	d = 0.22 (CCMI) d = 0.28 (ACP)	NA
**Bushey et al (2022**)	BPI total score (6 mo)	−0.46 (95% CI, −0.94 to 0.11)	.07
	BPI intensity (6 mo)	−0.53 (95% CI, −0.95 to −0.03)	.01
	BPI Interference (6 mo)	−0.39 (95% CI, −1.00 to 0.21)	0.14
	BPI total score (12 mo)	−0.54 (95% CI, −1.18 to −0.31)	.04
	BPI intensity (12 mo)	-0.62 (95% CI, −1.19 to −0.16)	.004
	BPI interference (12 mo)	-0.48 (95% CI, −1.25 to 0.04)	.09
**Darnall et al 2018**	Pain intensity	–	.29
	Pain interference	–	.44
**DeBar et al (2022**)	Pain impact (PEGS) 12 mo	–0.434 (95% CI, −0.690 to −0.178)	NA
	Pain impact (PEGS) posttreatment	−0.565 (95% CI, −0.796 to −0.333)	NA
**Garland et al (2019**)	BPI pain severity (Posttreatment)	−0.60 (−1.14, −0.07)	.031
**Garland et al (2022**)	BPI pain severity	0.49 (95% CI, 0.17–0.81)	.003
	BPI pain interference	1.07 (95%CI, 0.64–1.50)	*P* < .001
**Mehl-Madrona et al (2016**)	Pain intensity	NA	*P* = .01
**Sandhu et al (2023**)	Pain interference (PROMIS-PI-SF-8a) at 12 mo	−0.52 (95% CI, −1.94 to 0.89)	.15
	Pain intensity (PROMIS-PI-SF-3a)	−0.91 (95%CI, −2.64 to 0.83)	.10
**Sullivan et al (2017**)	BPI pain interference	−1.39 (95% CI, −2.78, −0.01)	.049
**Wilson et al (2023**)	BPI pain intensity	−0.09 point (95% CI, −0.29, 0.12)	.38
	BPI pain interference	−0.14 point (95% CI, −0.34, 0.07)	.23

ACP = attention control with psychoeducation, BPI = brief pain inventory, CCMI = collaborative care with motivational interviewing, CI = confidence interval, NA = not available, PEGS = pain, enjoyment of Life and general activity scale, PROMIS-PI-SF = patient-reported outcomes measurement information system pain interference short form.

### 3.8. Pain catastrophising

Only 2 studies measured Pain Catastrophising, Bushey^[54]^ used the Pain Catastrophizing Scale, with no significant differences observed between the intervention group and the control group at 12 months follow-up (95% confidence interval [CI], −1.79 to 5.210; *P* = .15). However, Darnall^[68]^ reported significant reductions in Pain Catastrophizing Scale between the beginning and end of the intervention [median 22 (IQR 10–30) - median 15 (IQR 7–23), *P* = .04].

### 3.9. Pain self-Efficacy

All 3 studies that measured Pain Self-Efficacy used the Pain Self-Efficacy Questionnaire and showed significant improvements at the end of the intervention. However, these improvements were not sustained at follow-up. In Sandhu,^[65]^ after 4 months, the intervention group showed significant improvements in pain self-efficacy in comparison with the usual care group (mean difference 2.39; 95% CI: −0.78 to 5.56, *P* < .001); however, these differences were not achieved between groups at eight and twelve months of follow-up. Similarly, Sullivan^[69]^ reported significant improvements in self-efficacy for patients in the taper-with-support group in comparison with the usual care group at 22 weeks [adjusted mean difference 7.86 (95% CI: 1.22, 14.50; *P* = .02)]. However, differences between groups were not significant at 34 weeks. Also, Wilson^[70]^ found a significant improvement in the E-health group compared to the usual care group at the end of treatment, though follow-up was not included in this study.

### 3.10. Pain-related disability

Two studies measured Pain-related Disability and used the Roland-Morris Disability Questionnaire. One study^[73]^ reported significant reductions between groups from baseline to after intervention and at 12-month follow-up. However, in the other study^[54]^ there were no significant differences between groups.

### 3.11. QoL and physical health

Four studies measured QoL and physical health with mixed results. Bushey^[54]^ used the Short Form Health Survey 36–Items (36-item Short Form Health Survey General Health) to measure QoL among their sample and found no significant differences between the intervention group and the control group. Similarly, Wilson^[70]^ used the Patient-Reported Outcomes Measurement Information System global physical and mental health measures to evaluate QoL and physical and mental health. Neither the physical health scores nor the mental health scores showed a significant difference. Mehl-Madrona^[74]^ used the Measure Yourself Medical Outcome Profile measure and showed no significant change regarding activities of daily living. Conversely, the QoL scores in the Measure Yourself Medical Outcome Profile showed significant improvements from before to after the intervention (−1.42 (95% CI −0.59 to −1.62), [*P* = .007]). Also, Sandhu^[65]^ used the EuroQol 5-dimension 5-level utility and the EuroQol 5-dimension 5-level visual analog scale to measure overall QoL and perception of health-related QoL, respectively. The study reported significant improvements at 4 months follow-up for the intervention group in both overall QoL [mean difference 0.03 (95% CI: −0.03 to 0.08), and the adjusted effect estimate was 0.57 (95% CI: 0.01 to 0.10) (*P* = .02)] and health-related perception of QoL [mean difference 1.66 (95% CI: −2.72–6.04), and the adjusted effect estimate was 4.43 (95% CI: 0.70 to 8.16) (*P* = .02)].

### 3.12. Emotional distress

Two studies measured Emotional Distress. Garland^[71]^ used the Depression Anxiety Stress Scale, version 21 and they reported larger reductions in the intervention group in emotional distress in comparison with the control group (2.79 points [95% CI, 0.41–5.18 points; *P* = .02]). Similarly, Sandhu^[65]^ used the Hospital Anxiety and Depression Scale (HADS) depression subscale and the 12-item Short Form Health Survey mental health component. At 4-month follow-up, the intervention participants experienced improvements in mental health and a reduction in emotional distress reported by higher scores in the 12-item Short Form Health Survey and lower scores in the HADS. Likewise, the mean difference in scores in the HADS depression subscale between the intervention group and the usual care was −0.55 (95% CI: −1.53 to 0.42; (*P* = .02).

### 3.13. Opioid craving

Only Garland^[71]^ measured opioid craving during the intervention; the study used the ecological momentary assessments of opioid craving on a scale of 0 to 10 (a higher score implying higher craving). The study showed that the intervention group had a significantly larger reduction in opioid craving scores in comparison with the control group (−0.49 points [95% CI, 0.27–0.70 points; *P* < .001]).

### 3.14. Opioid withdrawal symptoms

Sandhu^[65]^ used the Severity of Opioid Withdrawal Scale to measure opioid withdrawal symptoms among participants. There were no significant differences between the intervention group and the control group (mean difference at 4-month follow-up −0.4 [95%CI, −1.59–0.79], *P* = .18).

### 3.15. Opioid difficulties

Sullivan^[69]^ used the Prescription Opioid Difficulties Scale to assess problems related to opioid use and tapering among their sample. The study reported that at 22 weeks, the participants in the taper group had significantly lower scores in the Prescription Opioid Difficulties Scale compared to the usual care group, demonstrating fewer problems in tapering and opioid use [adjusted mean difference was (−4.90 (95% CI: −8.40, −0.80; *P* = .02). However, this difference was no longer significant at 34 weeks (adjusted mean difference was [−4.74 (95% CI: 10.13, 0.64; *P* = .08]).

### 3.16. Sleep quality

Only one study,^[65]^ measured Sleep Quality, and they used the Pittsburgh Sleep Quality Index. The participants’ scores did not differ significantly between the intervention group and the usual care group at 4 months nor at 8 and 12 months follow-up, showing similar results in terms of sleep quality for both groups.

### 3.17. Coping strategies

Coping strategies were measured by one study ^[70]^ and used the Coping Strategies Questionnaire-Revised. After 10 months, the intervention group significantly improved their coping strategies compared to the usual care group, specifically the subscales of catastrophizing: X2(1) = 4.50, *P* = .03; distraction: X2(1) = 9.22, *P* = .002; passive coping: X2(1) = 4.86, *P* = .03. The largest effect size is reflected in the catastrophizing subscale, with a Cohen’s *d* of 0.30.

### 3.18. BCTs used

The most used BCTs across all the interventions were Credible source, Information about health consequences, Instruction on how to perform the behavior, Goal setting (outcome). The most used group of BCTs were Goals and Planning and Shaping Knowledge. The intervention studies mentioned in this review used between eight^[68]^ and 34^[65]^ BCTs each (Table S3, Supplemental Digital Content 5).

### 3.19. BCTs linked with effective studies

The studies that showed significant changes in opioid dosage^[65–68,71,72,74]^ the group of BCTs mostly used were Goals and Planning, Shaping Knowledge, Natural Consequences, Repetition and Substitution, Regulation (reduce negative emotions) and Antecedents (Table S4, Supplemental Digital Content 6).

Two studies showed improvements in Opioid Misuse Risk^[71,72]^, and the BCTs used in these studies were Feedback and Monitoring, Shaping Knowledge, Natural Consequences, Comparison of Behaviour, Repetition and Substitution, Comparison of Outcomes, Regulation, Antecedents and Identity (Table S5, Supplemental Digital Content 7). Studies that showed improvements in Pain Impact Outcomes^[54,69,71–74]^ frequently used Goals and Planning; Shaping Knowledge; Natural Consequences; Repetition and Substitution; and Antecedents techniques in their intervention studies (Table S6, Supplemental Digital Content 8).

The 3 studies that showed pain self-efficacy improvements^[65,69,70]^ used most often behavioral change techniques such as Goals and Planning; Shaping Knowledge; and Antecedents (Table S7, Supplemental Digital Content 9).

## 4. Discussion

The purpose of this review was to evaluate BCTs for CNCP that were implemented in primary care settings and measure their effectiveness in reducing opioid use. Thirteen studies met the eligibility criteria; however, the studies were heterogeneous mainly regarding intervention design, delivery, participant eligibility criteria, enrollment dosage criteria and taper protocol. Additionally, different methods were used to report opioid taper and adherence rates. While some of the interventions did not specifically target opioid reduction, the main goal was to modify patient behavior, reduce opioids or better manage pain. In relation to opioid dosage reduction, the studies largely reported success. Six studies reported statistically significant reductions between treatment groups after the intervention; of those, one was of strong quality^[71]^ and one of moderate quality.^[70]^ The remaining 4 studies were of weak quality.^[65,67,72,74]^ Two other studies that did not include a comparator group reported significant reductions in opioid dosage pre- and post-intervention,^[66,68];^ these studies were also considered of weak quality.

Regarding the diversity of psychosocial interventions examined, various studies employed distinct approaches including CBT, mindfulness, and psychoeducation (see Table S1, Supplemental Digital Content 2). Of note, most of the studies that were statistically significant in tapering opioid medication included mindfulness approaches and techniques. This suggests a potential relationship between mindfulness and the observed reductions in opioid use. Mindfulness practices emphasize self-awareness, emotional regulation, and non-reactivity to pain. This approach may help individuals to navigate the complexities of CNCP while reducing reliance on opioids.^[77]^

This relationship is further examined through systematic mapping of the thirteen studies to the BCT Taxonomy. The studies were mapped to 58 of the 93 possible BCTs. The studies used between 8^[54,78]^ and 34 BCTs^[65]^ per study. Overall, the BCTs used more frequently were *Credible source* (n = 11); *Information about Health Consequences* (n = 11), *Goal Setting (outcome*) (n = 10), *Instruction on How to perform the behavior* (n = 10), *Reduce negative emotions* (n = 9), *Distraction* (n = 9), *Body Changes* (n = 9) and *Framing/Reframing* (n = 9). In relation to effective interventions to reduce opioids in CNCP, the eight studies that showed positive results in reducing opioid prescription, the groups of BCTs frequently used by these studies were *Goals and Planning, Shaping Knowledge, Natural Consequences, Repetition and Substitution, Regulation* and *Antecedents*. In addition, the BCTs that were used in most of the effective studies were *Goal Setting (Outcome)* (n* = 6), Instructions in How to perform the Behaviour* (n* = 7), Information about health consequences* (n* = 7), Behaviour Substitution* (n* = 6), Credible Source* (n* = 6), Reduced Negative emotions* (n* = 7), Distraction* (n* = 7), Body Changes* (n* = 7) and Framing/Reframing* (n* = 7*). These were the same BCTs used by most of the reviewed studies except Behaviour *Substitution*. This BCT was used in 6 of the effective studies in reducing opioids and one other noneffective study.^[73]^ These results suggest that *Behaviour Substitution* techniques may have played a role in supporting behavior change for reducing opioids in CNCP. *Behaviour Substitution* is a technique that may be helpful in interventions that aim to reduce, remove or replace a behavior. As described by Michie and colleagues,^[41]^ it facilitates replacing the undesired behavior by desirable or neutral behavior, therefore decreasing the frequency of an undesired behavior and increasing the frequency of a desired behavior (BCT Taxonomy v1). An advantage of employing this technique is that it enables the patient to redirect their focus towards adopting new behaviors rather than just removing the reliance on opioid medication. The use of *Behavioural Substitution* in other interventions has been corroborated across existing literature. A review of BCTs used in implementation and de-implementation interventions of healthcare practices,^[79]^ found that *Behaviour Substitution* was used more frequently in de-implementation interventions than implementation interventions. This study considers de-implementation interventions, interventions that aim to decrease ineffective or harmful behaviors. Furthermore, a review by Michie and colleagues identified that *Behavioural Substitution* only appeared in brief alcohol interventions but not in other behavioral interventions to change behavior.^[80]^ This highlights a shared direction on reducing or removing existing behaviors, alcohol consumption or opioid use for CNCP. This similarity suggests a common thread in stopping or replacing existing unhealthy behaviors and suggests that interventions to stop a behavior may require a different design compared to interventions to start a new behavior.

Furthermore, a systematic review and meta-analysis on interventions to deprescribe medication found similar groups of BCTs that were effective in reducing medication and deprescribing, including *goals and planning; shaping knowledge; natural consequences; regulation and antecedents*. However, they also found that the groups of BCTs, such as *Social Support, Comparison of the behavior, comparison of outcomes and Identity* were found to be effective in those trials.^[81]^ Another systematic review on interventions for medication adherence and illness beliefs for chronic conditions found that *Information about health consequences* was the most frequently used BCTs and similarly found that *Instructions on how to perform the behavior* were used in most of the interventions that targeted adherence outcomes. The authors also found that *Salience of Consequences*, *Biofeedback*, and *Social support* were frequently used in effective interventions.^[82]^ The interventions examined in the reviews mentioned above share similarities with trials aimed to taper opioids in CNCP, as these target the same behavior change goals and objectives as interventions to manage medication adherence and medication deprescribing. In relation to pain impact, six of the studies reported in this review were shown to be effective in improving pain impact outcomes. The BCTs used in more than half of the studies were *Instruction on how to perform the behavior, information about antecedents, reattribution, Information about health consequences, social comparison, Behavioural Practice/rehearsal, Credible Source, reducing negative emotions, Distraction, Body changes and Framing/reframing*. Previous research investigating BCTs used in self-management programmes for chronic low back pain and arthritis also recognized the use of techniques such as *Instruction on how to perform the behavior*, *Demonstration of the behavior, behavioral practice, credible source, body changes and graded tasks*.^[83]^ Moreover, a systematic review of BCTs to improve adherence to physical activity in musculoskeletal pain also found that *Goal Setting and Instruction on how to perform the Behaviour* were among the most used BCTs in effective studies. Also, the review reported that *Demonstration of the Behaviour, Practice/rehearsal and Social support* also improved exercise adherence for this patient group.^[84]^ Moreover, another review to improve adherence to physical exercise in musculoskeletal pain reported *graded tasks*, *goal setting (behavior*), *self‐monitoring of behavior*, *problem-solving* and feedback on behavior as the BCTs found to be used in interventions to promote physical activity in patients with chronic musculoskeletal conditions.^[85]^ Likewise, systematic reviews of interventions for pain management share similarities with interventions to reduce opioids in CNCP, as the participant sample will share similar characteristics and the goal of these interventions is to improve pain management with these patients.

In conclusion, this systematic review provides a novel and comprehensive analysis of the literature about psychosocial interventions to taper opioids in CNCP in primary care. The complexity of this topic is highlighted by the multitude of approaches used in these studies, including CBT, psychoeducation, nurse care management and mindfulness. Of note is the frequency with which some BCTs can be associated with positive outcomes, in particular the occurrence of the BCT B*ehaviour Substitution* for opioid dosage outcome. This review distinguishes the components and BCTs frequent to interventions targeting opioid reduction for CNCP in primary care.

Furthermore, these findings suggest that elements of mindfulness and behavior substitution techniques should be used in interventions to reduce opioids in CNCP providing guidance for research in this area. Nevertheless, these findings need to be interpreted carefully as the present study exhibits several weaknesses. High heterogeneity in the design of the interventions made a comparison between trials difficult, and the inclusion of trials of poorer quality might have overstated the effectiveness of the trials. Furthermore, the process of mapping the BCTs to the taxonomy presented some challenges. As it is only possible to code the BCTs if they are adequately described in the study, and some studies provided limited detail, some trials might likely have used additional BCTs that could not be detected by the researchers. To reduce the chances of mistakes and coding errors, the authors of the chosen studies were contacted for additional information on the interventions. Six authors responded and provided more details that helped in mapping the techniques used, such as supplementary materials and clarification of intervention components, which helped in mapping the techniques used. Where no response was received, BCT coding was based on the published article and any available supplementary materials. Yet the purpose of the BCT Taxonomy is to provide a tool for consistent identification of techniques used in different trial designs.

This systematic review draws attention to the most used BCTs in interventions to reduce opioids in CNCP to date. Furthermore, we suggest that applying the underlying principles of mindfulness and techniques such as goal setting, instructions on how to perform the behavior, information about health consequences, credible source, reduce negative emotions, distraction, body changes, framing/reframing and behavior substitution can offer a clear structure and increase the effectiveness of this type of interventions.

Additional empirical work is required to verify the connections between these BCTs and the withdrawal of opioids in CNCP.

## Acknowledgments

The authors would like to thank the University librarian, Carolyn Benny, for her valuable support in reviewing the data search strategy.

## Author contributions

**Conceptualization:** Andreia Ramos Silva, Catharine Montgomery, Bernhard Frank, Helen Poole.

**Data curation:** Andreia Ramos Silva.

**Formal analysis:** Andreia Ramos Silva.

**Investigation:** Catharine Montgomery, Katie Herron, Helen Poole.

**Methodology:** Catharine Montgomery, Katie Herron, Helen Poole.

**Supervision:** Catharine Montgomery, Bernhard Frank, Katie Herron, Helen Poole.

**Writing – original draft:** Andreia Ramos Silva.

**Writing – review & editing:** Andreia Ramos Silva, Catharine Montgomery, Bernhard Frank, Katie Herron, Helen Poole.


















