# Supplementary material for: Behaviour change interventions to reduce opioids in chronic non-cancer pain in primary care: A systematic review and mapping of behavior change techniques
Source: Medicine (Baltimore). 2026 Aug 7;105(32):e50106. doi: 10.1097/MD.0000000000050106 (PMC13456882; doi:10.1097/MD.0000000000050106)
Supplement: Supplementary file 1 [file medi-105-e50106-s001.docx]

S1 Search Strategy

**Behaviour change interventions to reduce opioids in chronic non-cancer pain in primary care: a systematic review and mapping of behaviour change techniques**

Andreia Ramos Silva, Catharine Montgomery, Bernhard Frank, Katie Herron, Helen Poole

Search Terms

| Concept | Text words | Controlled vocabulary (MESH terms) |
| --- | --- | --- |
| Chronic non-cancer pain | Chronic pain | Chronic pain |
|  | Pain |  |
|  | Headache |  |
|  | migraine |  |
|  | Sciatica |  |
|  | Backache |  |
|  | arthritis |  |
|  | Rheumatoid arthritis |  |
|  | Osteoarthritis |  |
|  | Neuralgia |  |
|  | Fibromyalgia |  |
|  | Back pain |  |
|  | Musculoskeletal pain |  |
|  | Pelvic pain |  |
|  | Neuropathic pain |  |
|  |  |  |
|  |  |  |
| Opioids | Opioid |  |
|  | opioids |  |
|  | opiate |  |
|  | analgesic |  |
|  | analgesics |  |
|  | Narcotic |  |
|  | narcotics |  |
|  | oxymorphone |  |
|  | Morphine |  |
|  | Buprenorphine |  |
|  | Fentanyl |  |
|  | methadone |  |
|  | oxycodone |  |
|  | Codeine |  |
|  | Hydrocodone |  |
|  | Meperidine |  |
|  | Tapentadol |  |
|  |  |  |
|  |  |  |
|  |  |  |
|  |  |  |
| Behavioural intervention | Psychotherapy |  |
|  | counselling |  |
|  | Coping |  |
|  | biofeedback |  |
|  | Therapy |  |
|  | “Motivational interviewing” |  |
|  | MI |  |
|  | “Therapeutic interactive voice response” |  |
|  | “Cognitive Behavioural intervention” |  |
|  | CBT |  |
|  | Acceptance and commitment therapy |  |
|  | ACT |  |
|  | interdisciplinary |  |
|  | Inter-disciplinary |  |
|  | Multi-disciplinary |  |
|  | multidisciplinary |  |
|  | Adaptive behaviour |  |
|  | Behaviour |  |
|  | Behavior |  |
|  | Education |  |
|  | Pain management |  |
|  | Mindfulness |  |
|  | Mindfulness-based |  |
|  | meditation |  |
|  |  |  |
| Primary care | Primary care |  |
|  | GP practices |  |
|  | Community practices |  |
|  | pharmacy |  |
|  |  |  |
|  |  |  |
| Reduction opioids | Deprescribing |  |
|  | Wean |  |
|  | Weaning |  |
|  | Reduction |  |
|  | Stop |  |
|  | Cessation |  |
|  | Ceas* |  |
|  | Taper |  |
|  | Tapering |  |
|  | Discontin* |  |
|  | Abstain* |  |
|  | Abstinen* |  |
|  | Detox* |  |
|  | Remove* |  |
|  | Terminant* |  |
|  | Substitu* |  |
|  | Withdrawal |  |
|  |  |  |
|  |  |  |

|  | Search words | PICO (T) or PCC |
| --- | --- | --- |
| 1 | pain OR “CNCP” OR headache OR migraine OR sciatica OR backache OR arthritis OR osteoarthritis OR neuralgia OR fibromyalgia | Chronic pain – Population |
| 2 | opioid* OR opiate OR analgesi* OR narcotic* OR oxymorphone OR morphine OR buprenorphine OR fentanyl OR methadone OR oxycodone OR codeine OR hydrocodone OR tapentadol OR meperidine OR pethidine OR demerol OR sufentanil | Intervention |
| 3 | deprescrib* OR withdraw* OR wean* OR reduc* OR stop* OR cessation OR ceas* OR taper* OR discontinu* OR abstain* OR abstinen* OR remov* OR terminat* OR substitu* | Outcome - concep |
| 4 | psychotherap* OR psychosocial OR counsel* OR coping OR therap*OR psychol* OR “motivational interviewing” OR “MI” OR “therapeutic interactive voice response” OR TIVR OR “behavio* intervention” OR “CBT” OR “acceptance and commitment therap*” OR “ACT” OR interdisciplinary OR multidisciplinary OR “behavio* change” OR “behavio* strateg*” OR “behavio* skill*” OR education* OR mindfulness* OR “pain management” OR meditation OR patient-led OR patient-centered OR patient-centred OR person-centered OR person-centred OR person-focused OR patient-focused | Behavioural intervention- therapy – Intervention |
| 5 | “primary care” OR “general practic*” OR “community practic*” OR pharmac*OR “community pharmac*” OR “GP” OR “family practic*” | context- Primary care |

Medline ebsco

Filter: Humans, only english language, from 2021 until date, adults- 61results

Cinahl: 17 results.

Scopus: 4 results

( TITLE-ABS-KEY ( pain OR "CNCP" OR headache OR migraine OR sciatica OR backache OR arthritis OR osteoarthritis OR neuralgia OR fibromyalgia ) AND TITLE-ABS-KEY ( opioid* OR opiate OR analgesi* OR narcotic* OR oxymorphone OR morphine OR buprenorphine OR fentanyl OR methadone OR oxycodone OR codeine OR hydrocodone OR tapentadol OR meperidine OR pethidine OR demerol OR sufentanil ) AND TITLE-ABS-KEY ( deprescrib* OR withdraw* OR wean* OR reduc* OR stop* OR cessation OR ceas* OR taper* OR discontinu* OR abstain* OR abstinen* OR remov* OR terminat* OR substitu* ) AND TITLE-ABS-KEY ( psychotherap* OR psychosocial OR counsel* OR coping OR therap*or AND psychol* OR "motivational interviewing" OR "MI" OR "therapeutic interactive voice response" OR tivr OR "behavio* intervention" OR "CBT" OR "acceptance and commitment therap*" OR "ACT" OR interdisciplinary OR multidisciplinary OR "behavio* change" OR "behavio* strateg*" OR "behavio* skill*" OR education* OR mindfulness* OR "pain management" OR meditation OR patient-led OR patient-centered OR patient-centred OR person-centered OR person-centred OR person-focused OR patient-focused ) AND TITLE-ABS-KEY ( "primary care" OR "general practic*" OR "community practic*" OR pharmac*or "community pharmac*" OR "GP" OR "family practic*" ) ) AND ( LIMIT-TO ( PUBYEAR , 2023 ) OR LIMIT-TO ( PUBYEAR , 2021 ) ) AND ( LIMIT-TO ( LANGUAGE , "English" ) )

References for results in scopus:

Guillou Landreat, M., Baillot, M., Le Goff, D., & Le Reste, J. Y. (2021). Acute pain management among patients with opioid maintenance therapy: Specificities and difficulties identified in primary care: A qualitative study. BMJ Open, 11(1) doi:10.1136/bmjopen-2020-044433

WEB of Science: results 63

Search strategy:

\# Web of Science Search Strategy (v0.1)

Search: pain OR “CNCP” OR headache OR migraine OR sciatica OR backache OR arthritis OR osteoarthritis OR neuralgia OR fibromyalgia (Title) OR pain OR “CNCP” OR headache OR migraine OR sciatica OR backache OR arthritis OR osteoarthritis OR neuralgia OR fibromyalgia (Abstract)	Date Run: Wed May 31 2023 13:02:59 GMT+0100 (British Summer Time)	Results: 1066036

Search: opioid* OR opiate OR analgesi* OR narcotic* OR oxymorphone OR morphine OR buprenorphine OR fentanyl OR methadone OR oxycodone OR codeine OR hydrocodone OR tapentadol OR meperidine OR pethidine OR demerol OR sufentanil (Title) OR opioid* OR opiate OR analgesi* OR narcotic* OR oxymorphone OR morphine OR buprenorphine OR fentanyl OR methadone OR oxycodone OR codeine OR hydrocodone OR tapentadol OR meperidine OR pethidine OR demerol OR sufentanil (Abstract)	Date Run: Wed May 31 2023 13:06:57 GMT+0100 (British Summer Time)	Results: 285940

Search: deprescrib* OR withdraw* OR wean* OR reduc* OR stop* OR cessation OR ceas* OR taper* OR discontinu* OR abstain* OR abstinen* OR remov* OR terminat* OR substitu* (Title) OR deprescrib* OR withdraw* OR wean* OR reduc* OR stop* OR cessation OR ceas* OR taper* OR discontinu* OR abstain* OR abstinen* OR remov* OR terminat* OR substitu* (Abstract)	Date Run: Wed May 31 2023 13:09:07 GMT+0100 (British Summer Time)	Results: 9761047

Search: psychotherap* OR psychosocial OR counsel* OR coping OR therap*OR psychol* OR “motivational interviewing” OR “MI” OR “therapeutic interactive voice response” OR TIVR OR “behavio* intervention” OR “CBT” OR “acceptance and commitment therap*” OR “ACT” OR interdisciplinary OR multidisciplinary OR “behavio* change” OR “behavio* strateg*” OR “behavio* skill*” OR education* OR mindfulness* OR “pain management” OR meditation OR patient-led OR patient-centered OR patient-centred OR person-centered OR person-centred OR person-focused OR patient-focused (Title) OR psychotherap* OR psychosocial OR counsel* OR coping OR therap*OR psychol* OR “motivational interviewing” OR “MI” OR “therapeutic interactive voice response” OR TIVR OR “behavio* intervention” OR “CBT” OR “acceptance and commitment therap*” OR “ACT” OR interdisciplinary OR multidisciplinary OR “behavio* change” OR “behavio* strateg*” OR “behavio* skill*” OR education* OR mindfulness* OR “pain management” OR meditation OR patient-led OR patient-centered OR patient-centred OR person-centered OR person-centred OR person-focused OR patient-focused (Abstract)	Date Run: Wed May 31 2023 13:09:56 GMT+0100 (British Summer Time)	Results: 2767793

Search: “primary care” OR “general practic*” OR “community practic*” OR pharmac*OR “community pharmac*” OR “GP” OR “family practic*” (Title) OR “primary care” OR “general practic*” OR “community practic*” OR pharmac*OR “community pharmac*” OR “GP” OR “family practic*” (Abstract)	Date Run: Wed May 31 2023 13:12:32 GMT+0100 (British Summer Time)	Results: 221417

Search: #1 AND #2 AND #3 AND #4 AND #5	Date Run: Wed May 31 2023 13:13:25 GMT+0100 (British Summer Time)	Results: 243

Search: #1 AND #2 AND #3 AND #4 AND #5	Date Run: Wed May 31 2023 13:13:58 GMT+0100 (British Summer Time)	Results: 243

Search: #1 AND #2 AND #3 AND #4 AND #5 and 2021 or 2022 or 2023 (Publication Years)	Date Run: Wed May 31 2023 13:14:40 GMT+0100 (British Summer Time)	Results: 63

\# Database: Web of Science Core Collection

\# Entitlements:

- WOS.IC: 1993 to 2023

- WOS.CCR: 1985 to 2023

- WOS.SCI: 1970 to 2023

- WOS.AHCI: 1975 to 2023

- WOS.BHCI: 2005 to 2023

- WOS.BSCI: 2005 to 2023

- WOS.ESCI: 2015 to 2023

- WOS.ISTP: 1990 to 2023

- WOS.SSCI: 1970 to 2023

- WOS.ISSHP: 1990 to 2023

\# Searches:

Search: #1 AND #2 AND #3 AND #4 AND #5 and 2021 or 2022 or 2023 (Publication Years) and English (Languages)	Date Run: Wed May 31 2023 13:15:06 GMT+0100 (British Summer Time)	Results: 63

Total results in the 4 databases: 145- 38 duplicates= 107
