# Supplementary material for: Behaviour change interventions to reduce opioids in chronic non-cancer pain in primary care: A systematic review and mapping of behavior change techniques
Source: Medicine (Baltimore). 2026 Aug 7;105(32):e50106. doi: 10.1097/MD.0000000000050106 (PMC13456882; doi:10.1097/MD.0000000000050106)
Supplement: Supplementary file 2 [file medi-105-e50106-s002.docx]

S1 Table: Study Characteristics

**Behaviour change interventions to reduce opioids in chronic non-cancer pain in primary care: a systematic review and mapping of behaviour change techniques**

Andreia Ramos Silva, Catharine Montgomery, Bernhard Frank, Katie Herron, Helen Poole

| Author, Year and Country | Study Design | Population | Completers or dropouts | Patient characteristics at baseline | Intervention description including duration and frequency | Staff delivering the int., and follow-up | Comparator | Primary outcomes | Secondary Outcomes |
| --- | --- | --- | --- | --- | --- | --- | --- | --- | --- |
| Borsari et al., 2021; USA | RCT | n=100, CCMI=51 and ACP=49 veterans with chronic pain > 6 months duration, prescribed opioids at least 90 days and at high-risk opioid use | 74% of the participants completed the primary care study visits. | Veterans, in the CCMI group, mean age 63.2 (SD 10.6) and in the ACP group was 62.6 (SD 7.1).  CCMI group 92.2% were male and in the ACP group, 87.8% were male. | Collaborative care with Motivational interviewing (CCMI).  1-hour visit before enrolment and then 4 sessions of 20-30 minutes (4, 6, 8 and 12 weeks) and then 30-minute follow-up sessions at 20 weeks. | Primary care physician and psychologist or psychology research staff trained in MI | Attention control with psychoeducation (ACP).  Follow up at 20 weeks. | Opioid risk and pain interference | Current opioid misuse, pain severity, additional behaviours, and adoption of SMART goals for pain management. And opioid dosage changes as an exploratory outcome. |
| Bushey et al., 2022; USA | RCT | n=261, MED group =131 and CBT= 130 veterans with chronic low back pain prescribed opioids for at least 3 or more prescriptions of any dosage for 28 days or more during the last 12 months. | Dropouts: 29 participants (11.1%) leave the study (12 in the MED  group and 17 in the CBT group). | 241 [92.3%] men; mean [SD] age, 57.9 [9.5] years) | Collaborative care medication optimisation- Care Management for effective use of opioids (CAMEO), 1st consultation in person and then telephone consultations with Nurse 10-20 minutes. 8 weekly treatment sessions of approximately 30 minutes for 6 months and 1 check in session at 9 months | Trained nurses, 12 months | CBT | Pain intensity and pain interference | Pain intensity, pain interference, pain-related disability, pain catastrophising, self-reported substance misuse, health-related quality of life, depression and anxiety. Analgesic dose was collected as an exploratory outcome. |
| Darnall et al., 2018; USA | Single arm prospective cohort pre and post-design | n=82 patients with CNCP | Completers n=51 | Age: mean 51 (SD 12) years,  Sex: 41 participants  (60%) were female | Patient-centred opioid tapering programme including education, self-help book and gradual tapering. Duration: 4 months with session at least once a month. | No follow-up, primary care physician | Pre and post-intervention | Changes in MED from baseline to end of intervention | Pain intensity |
| DeBar et al., 2022; USA | RCT | n=850 CNCP patients. With at least 2 prescriptions of long-acting opioids in the last 6 months or a supply of short-acting opioids of at least 90 days for the last 4 months. | Completers: 816 (96.0%) completed follow-up assessments | mean age, 60.3 years; Sex: 67.4% women. | Interdisciplinary CBT teaching pain self-management skills. 3 months, 12 weekly sessions of 90 minutes. | Interdisciplinary team of behavioural health specialists, nurse care manager, physical therapist and pharmacist working in collaboration with primary care physician. Follow-up at 6 and 12 months | Usual care | Self-reported pain impact | Pain-related disability, satisfaction with care, opioids and benzodiazepines used (medical records) |
| Garland et al., 2020 and Garland et al., 2019  USA | RCT | n=95, CNCP patients taking opioids for at least 90 days. More n=50 and SG n=45. | Completers (attending at least 4 sessions): 80% (74% in the MORE group and 86% in the SG group) | Age: mean age 56.6 (SD1.21) years.  Sex: 66.3% female. | Mindfulness- Oriented Recovery Enhancement (MORE)- mindfulness, reappraisal and savouring positive experiences.  8 weekly 2 hours, plus daily practice of mindfulness | Clinical social worker and researchers (authors) | Active Support Group (SG) | Opioid dose and HRV (Heart Rate Variability) at meditation; Opioid Misuse Risk and Pain Severity | Not differentiated |
| Garland et al., 2022; USA | RCT | n=250 CNCP patients misusing prescription opioids | Dropout: 92 of 250  participants (36.8%) | Age: mean age,51.8 (SD 11.9) years  Sex: 159 women(63.6%) | Mindfulness- Oriented Recovery Enhancement (MORE)- mindfulness, reappraisal and savouring positive experiences.  8 weekly 2 hours, plus daily practice of mindfulness | Researchers (authors) clinical social workers and a nonauthor clinician provided intervention for both groups. Follow-up at 3, 6 and 9 months after intervention. | Supportive Group psychotherapy | Opioid misuse and pain severity and pain-related functional interference. | Opioid dose, emotional distress, opioid craving |
| Mehl-Madrona et al., 2016; USA | Quality improvement matched the prospective cohort | n=84 CNCP patients accepted GMVs, sample characteristics from 414 participants. 207 from the group visits and 207 from the matched cohort, although only data from 84 patients were used for data analysis. | Of the 84 patients included in the GMVs, 42 attended for at least 6 months, 37 dropouts. | Mean age in the group visits group was 45.7 (SD18.1) and in the treatment-as-usual group 44.3 (SD 13.1). Sex: 124 were women in the group visits and 124 in the treatment as-usual group | Psychoeducation, mindfulness, relaxation, and physical exercise. group visits. aim to reduce opioids. Group medical visits (GMVs) provided education about non-pharmacological methods for pain management and taught mindfulness techniques, movement, guided imagery, relaxation training, yoga, qigong, and t'ai chi. 12 required sessions of psychoeducation, 4 required sessions of physical activity per month, duration 6 months. | A doctor trained in behavioural health, a nurse and a behavioural health specialist. No follow-up. | Matched cohort for opioid dosage and pre-post test | Change in opioid dose morphine equivalents | Change in score in my Medical Outcome Profile and change in visual analogue pain ratings |
| Sandhu et al., 2023; UK | RCT | n=608 CNCP patients, median MED 46 mg. | 440 (72%) completed 12-month  follow-up and 47% of participants in the intervention group  completed  days 1 to 3 of group sessions. | Mean age, 61 years; 362 females [60%]; | Educational intervention and support with MI, including:  in person, phone calls and CDs and DVDs of mindfulness. 3-day group sessions, 1-hour individual session on MI, 230-minute phone calls and 1-hour face-to-face meeting. Totalling 7 hours of intervention during 8 to 10 weeks period. | Trained intervention nurse and a layperson with CNCP and experience in opioid tapering. Follow-up at 4, 8, 12 months. | Enhanced Usual Care | Pain -Reported Outcomes and the proportion of patients that discontinued opioids at 12 months (self-report). | Pain interference and self-reported opioid intake. The severity of opioid withdrawal, health-related quality of life, sleep quality and emotional well-being, |
| Scott et al., 2020; and Kesten et al., 2020  UK | Quality improvement, single-arm cohort | N=34 CNCP patients | Number of completers: 17 (50%). | Age: mean (SD) 51 (10),  Sex: 64.7% were female. | A personalised pain management plan including daily goals, relaxation, gentle exercises, coping with low mood, and adjusting sleep. Also, access to complementary care and support options were available, including physiotherapy and relaxation groups. Support in weaning opioids was provided if appropriate. The number and frequency of sessions were determined by the patient's needs and within the staff’ availability. | Project workers. No follow-up | Pre- and post-intervention | Opioid dose, current opioid misuse, pain interference, health, well-being and quality of life, healthcare use and delivery | No distinguishing from primary or secondary outcomes. |
| Seal et al., 2020, USA | Quality improvement matched the prospective cohort | n=294, 147 in the IPT group and 147 matched from usual care. veterans prescribed 90mg or more MEDD | Dropouts: not mentioned. | Age: mean in years in the IPT 62.1 (SD 12.4) and in the usual care 62.9 (SD 11.4).  Sex: 132 (89.8%) were male. | Integrated Pain Team (IPT) was a multidisciplinary pain care based on patients’ values and goals, emphasizing self-management strategies. IPT used behavioural health strategies (e.g., cognitive behavioural therapy for CNCP and mindfulness), physical modalities (e.g., acupuncture, chiropractic care, heat, and ice), and exercise (e.g., physical therapy, yoga, and Tai Chi).  4 visits of 30 minutes and 1 hour for the first one for 6 months | Interdisciplinary: doctor, psychologist, and pharmacist.  Follow-up at 6 months. | Compared two matched cohorts receiving chronic pain care. IPT (N = 147) versus usual primary care (UPC, N = 147) | Change in opioid dose in MED, from baseline, 3 months and 6 months. | Opioid risk mitigation. |
| Sullivan et al., 2017, USA | RCT | n=35, CNCP, daily MED >50 mg, willingness to taper opioid dose by at least 50% (or to 120 mg MED, whichever was less). | Dropouts: 3 | Mean (SD) age in years 54.4 (10.1). | Taper support intervention: including psychiatric meeting, opioid dose tapering, 18 weekly sessions with a doctor assistant to promote motivation for tapering and understanding of pain self-management. | Psychiatry pain medicine, physician, physician assistant. Follow-up at 34 weeks. | Usual Care | Change in opioid | Pain severity and pain activity-related interference, pain self-efficacy, depressive symptoms, opioid-related problems and concerns. |
| Vogler et al., 2017, USA | Single arm prospective cohort pre and post design | CNCP patients, n=35, opioid data collected from n=14 | Completers: 5 participants | Age: mean age 58 (SD 11) years, Sex: female 77% | Patient educational videos on CNCP delivered during patient groups and discussions after. 4 sessions of 90 minutes. | Primary care physician, nurse and pharmacist. No follow-up | Pre and post-intervention | Functional status | Knowledge assessment and chart review of opioid use and utilization of emergency and urgent care services. |
| Wilson et al., 2023, USA | RCT | n=402 patients with CNCP, at least on 20 mg MED. | Completers: 89% completed the 10-month follow-up | Mean Age (SD): 56.7 (11.0) years.  Sex: 279 women (69.4%) | E-health Goalistics Chronic Pain Management Program: online (videos) with offline activities with a 4-month subscription to the program. the e-health program could be finished within 8 weeks by doing 1 to 2 hours per week. | Developed by pain psychologists. Follow-up at 10 months. | Treatment as usual (TAU) | Yes or no to a reduction of 15% in opioids at 10 months. | The raw baseline and 10-month difference in MED, pain knowledge, pain self-efficacy, pain coping, intervention adherence |
