# Supplementary material for: Behaviour change interventions to reduce opioids in chronic non-cancer pain in primary care: A systematic review and mapping of behavior change techniques
Source: Medicine (Baltimore). 2026 Aug 7;105(32):e50106. doi: 10.1097/MD.0000000000050106 (PMC13456882; doi:10.1097/MD.0000000000050106)
Supplement: Supplementary file 3 [file medi-105-e50106-s003.docx]

S2 Table. Opioid Dosage Outcomes

| Study | Outcome Measure | Mean/ Median Baseline | Mean/Median  End of intervention or follow-up | Differences within group | Difference between groups | Tapering protocol |
| --- | --- | --- | --- | --- | --- | --- |
| Borsari et al., 2021 | Medical records | MEDD mean in the intervention group was 210.0 (SD 485.5) and the median (IQR) 52.5 (22.5-135) and control group ACP Mean was 143.8 (320.2 SD) and the Median 45 (17.5-125) | At 12 weeks MEDD mean 133.0 (292.8 SD) and Median 42.5 (21.3-73.3) in the intervention group, and 146.8 (317.1 SD) and Median 45 (20-142.5) in the control group | NA | Between baseline and 12 weeks a 27.5% (95%CI: 61.0% reduction to 6.0% increase) and 20 weeks 18.5% (95%CI: 21% reduction to 59% increase). Statistical significance was not achieved at 12 weeks (p=0.017) nor at 20 weeks (p=0.0348) | Did not have a tapering protocol or plan, up to the patient to reduce. |
| Bushey et al., 2022 | Medical records | MME for the MED group Mean (SD) 68.6 (81.0). For the CBT group mean 76.2 (96.5) | At 6 months in the MED group mean (SD)61.2 (75.2) and in the CBT group 72.7 (96.8). Data at 12 months in the MED group 54.1 (73.3) and the CBT group 66.7 (96.1) | NA | At 6 months: mean difference, −11.55 [95%CI, −32.73 to 9.62]; *p* = 0.28). At 12 months: mean difference, −12.53 [95%CI, −33.41 to 8.35]; *p* = 0.24 | Suggested tapering and occasional adjustment to opioid dosage (this intervention focuses on analgesic optimisation) |
| Darnall et al., 2018 | patient self-reported and medical records | MEDD median (IQR) 288 (153-587) | At 4 months MEDD median (IQR) 150 (54-248) | Before and after intervention: median of 150 mg (IQR: 54-248 mg) *p*=0.002 | NA | Included a personalised tapering plan |
| DeBAr et al., 2022 | Medical records | Median average daily dose of opioids was 29.6 MME | NA | NA | At  the posttreatment assessment (difference, -2.260 points [CI, -5.509 to 0.989 points]) or at 12 months (difference, -1.969 points [CI, -6.765 to 2.827 points]). No differences. | Did not include a taper protocol/plan |
| Garland et al., 2020 | Self-reported by interview (Timeline follow back assessment) and medical reports | In the MORE group, the MEDD mean 66.31 (SD 10.75mg ). In the SG group mean MED 69.65 (SD15.05) mg | At 3 months follow-up: in the MORE Group mean MED was 45.25 (SD10.08) mg . The SG group mean MED was 155.84 (SD 63.82) mg | At 3 months follow-up: ITT analysis conducted using linear mixed modelling, the main effects of neither group F(1, 92.68) =1.41, p = 0.24 nor time F(2, 129.77) = .99, p= 0.38, were statistically significant | At 3 months follow-up log transformed interaction group x time showed to be significant F(2, 129.77) =5.35, *p* = 0.006, d = 1.07. With the MORE group showed a significant decrease 31.8% in opioid dose and the SG had a 124.0% increase. With a sensitive analysis excluding 2 outliers of the SG group this remained statistically significant F(2, 59.92) = 4.41, p =0.016, d =0 .77 | Did not include a taper protocol/plan |
| Garland et al., 2022 | Self-report by interview Timeline Follow back assessment) | MEDD median probability (IQR) at baseline for MORE group 35.0 (15.0-80.0) and the Supportive Psychotherapy 38.0 (18.0-90.5) | MEDD posttreatment probability in the MORE group 24.0 (10.0-62.0) and the supportive psychotherapy 39.0 (18.5-83.5) | NA | Between-group effect: 0.15 log mg; 95%CI,0.03-0.27 log mg; P = .009) | Did not include a taper protocol/plan |
| Mehl-Madrona et al., 2016 | Medical records | Opioid reduction group mean (82.1mg MED (SD 21.0)), Conventional Care 72.1mg MED (21.7SD) | Opioid reduction group (32.4 mg MED (13.0 SD) and in conventional care 86.1 mg MED (21.5sd) | Statistical difference In the intervention group from baseline to end of treatment *p<* 0.001 | Statistical Difference between group between baseline and end of treatment p=0.005 | Did not have a tapering protocol. Tapering was not required but attendance to the programme required to continue to receive the same dosage prescription |
| Sandhu et al., 2023 | Self-reported | In the education and support group, Median (IQR) in mg was 49 (25-81) and In the usual care group 44 (25-75) | NA | NA | Difference between groups in reducing opioid dosage by at least 50% at 12 months was absolute difference, 29.9% [95% CI, 21.1% to 38.8%]; odds ratio, 3.76 [95%CI, 2.47 to5.71]; P < .001. Also at 12 months, the difference between groups in stopping opioids was absolute difference, 21.7% [95%CI, 14.8%to 28.6%]; P < .001; odds ratio, 5.55 [95% CI, 2.80 to 10.99] | Included individualised tapering plan |
| Scott et al., 2020 | Medical Records | Median 90 mg (IQR 60 to 240) | Median 72mg (IQR 30 to 160) | Significant differences from baseline to follow-up *p*<0.001 | NA | Suggested tapering protocol but not required. |
| Seal et al., 2020 | Medical records | Mean opioid dose (in MEDD) was 124.1mg (SD ± 241.1) in the IPT group and 124.5 (231.5) in the usual care group | Mean at 6 months in the IPT team 68.4 (166.1) and the usual care 107.1 (223.4) | NA | The mean opioid dose reduction was significantly greater in the IPT versus UPC group by 6 months *(p* < 0.03). | Included Individualised opioid taper (overseen by a pharmacist) |
| Sullivan et al., 2017 | Self-reported and medical records | Mean daily opioid dose was 207.2 mg MED (269.3 SD) in the taper with support group and 245.2mg (347.3 SD) in the usual care group. | The mean percent reduction from baseline in the opioid taper support group was 52%, while it was 31% in the usual care group. | NA | No statistically significant differences at 22 weeks, adjusted mean difference between groups (=-42.9mg MED; 95%CI: -92.42, 6.62; p=0.09) neither at 34 weeks was -26.7 mg MED (95% CI: -83.04, 29.62; p = 0.34) | Included tapering protocol of 10% reduction per week. |
| Vogler et al., 2017 | Self-reported and medical records | n=14, median MED 17.5 mg (range 0-120mg) | n=14, median MED, 12.5mg (IQR 0-110mg) at last study visit. | The difference was not statistically significant (*p*= 0.39) | NA | Did not have a tapering protocol |
| Wilson et al., 2023 | Medical records | MED mean (SD) in the E-health group was 48.1 (63.6) and MED ranged from 20.0-660.0. In the usual care group, the mean MED was 47.8 (53.4) and MED range from 20.0-548.0 | at 10months E Health MED mean was 49.8 (87.2) and in the TAU group 61.1 (98.4) | NA | Statistically significant differences between groups on achieving at least 15% reduction favouring the E-health group: odds ratio, 1.6 [95% CI, 1.1-2.3]; P =0.02) | Did not have a tapering protocol |

NA- Not available or Not Applicable
