# Supplementary material for: Behaviour change interventions to reduce opioids in chronic non-cancer pain in primary care: A systematic review and mapping of behavior change techniques
Source: Medicine (Baltimore). 2026 Aug 7;105(32):e50106. doi: 10.1097/MD.0000000000050106 (PMC13456882; doi:10.1097/MD.0000000000050106)
Supplement: Supplementary file 4 [file medi-105-e50106-s004.docx]

S2 File. Studies Quality - Risk of Bias

**Behaviour change interventions to reduce opioids in chronic non-cancer pain in primary care: a systematic review and mapping of behaviour change techniques**

Andreia Ramos Silva, Catharine Montgomery, Bernhard Frank, Katie Herron, Helen Poole

**The Effective Public Health Practice Project (EPHPP) checklist criteria for each study**

# STUDY: BORSARI et al., 2021

**COMPONENT RATINGS**

A) **SELECTION BIAS**

**(Q1) Are the individuals selected to participate in the study likely to be representative of the target population?**

1 Very likely

2 Somewhat likely –

3 Not likely - referred by GP, ‘mail and call’ and flyers

4 Can’t tell

**(Q2) What percentage of selected individuals agreed to participate?**

1 80 - 100% agreement

2 60 – 79% agreement

3 less than 60% agreement (n=534 declined, randomised n=100)

4 Not applicable

5 Can’t tell

**RATE THIS SECTION**

**STRONG 1**

**MODERATE 2**

**WEAK 3**

B) **STUDY DESIGN**

Indicate the study design

1 Randomized controlled trial - randomisation process is explained in the study.

2 Controlled clinical trial

3 Cohort analytic (two group pre + post)

4 Case-control

5 Cohort (one group pre + post (before and after))

6 Interrupted time series

7 Other specify ____________________________

8 Can’t tell

Was the study described as randomized? If NO, go to Component C.

No

Yes

If Yes, was the method of randomization described? (See dictionary)

No

Yes

If Yes, was the method appropriate?

YES. ‘the study coordinator and the data manager used standard software to block randomise participants to either CCMI or ACP (1:1 allocation ratio), stratified a prior on current and former drug or alcohol use disorder.’

**RATE THIS SECTION**

**STRONG 1**

**MODERATE 2**

**WEAK 3**

C) **CONFOUNDERS**

**(Q1) Were there important differences between groups prior to the intervention?**

1 Yes

2 No

3 Can’t tell

The following are examples of confounders:

1 Race

2 Sex

3 Marital status/family

4 Age

5 SES (income or class)

6 Education

7 Health status

8 Pre-intervention score on outcome measure

(**Q2) If yes, indicate the percentage of relevant confounders that were controlled (either in the design (e.g. stratification, matching) or analysis)?**

1 80 – 100% (most)

2 60 – 79% (some)

3 Less than 60% (few or none)

4 Can’t Tell

The groups were stratified based on drug and alcohol use prior to randomisation. However, socio-demographics data at baseline shows major differences between groups for post-traumatic disorder.

**RATE THIS SECTION**

**STRONG 1**

**MODERATE 2**

**WEAK 3**

D) **BLINDING**

**(Q1) Was (were) the outcome assessor(s) aware of the intervention or exposure status of participants?**

1 Yes

2 No

3 Can’t tell

**(Q2) Were the study participants aware of the research question?**

1 Yes

2 No

3 Can’t tell

**RATE THIS SECTION**

**STRONG 1**

**MODERATE 2**

**WEAK 3**

E) **DATA COLLECTION METHODS**

**(Q1) Were data collection tools shown to be valid?**

1 Yes

2 No

3 Can’t tell

**(Q2) Were data collection tools shown to be reliable?**

1 Yes

2 No

3 Can’t tell

**RATE THIS SECTION**

**STRONG 1** measures for primary outcomes were COMM and BPI which are both valid and reliable measures, (α = 0.87) for this sample.

**MODERATE 2**

**WEAK 3**

F) **WITHDRAWALS AND DROP-OUTS**

**(Q1) Were withdrawals and drop-outs reported in terms of numbers and/or reasons per group?**

1 Yes

2 No

3 Can’t tell

4 Not Applicable (i.e. one time surveys or interviews)

**(Q2) Indicate the percentage of participants completing the study. (If the percentage differs by groups, record the lowest).**

1 80 -100%

2 60 - 79% , approximately 74% of enrolled veterans completed their PCP visits and 4 care manager contacts’

3 less than 60%

4 Can’t tell

5 Not Applicable (i.e. Retrospective case-control)

**RATE THIS SECTION**

**STRONG 1**

**MODERATE 2**

**WEAK 3**

**Not Applicable**

G) **INTERVENTION INTEGRITY**

**(Q1) What percentage of participants received the allocated intervention or exposure of interest?**

1 80 -100%

2 60 - 79% ‘approximately 74% of enrolled veterans completed their PCP visits and 4 care manager contacts’

3 less than 60%

4 Can’t tell

**(Q2) Was the consistency of the intervention measured?**

1 Yes fidelity monitoring of the sessions.

2 No

3 Can’t tell

**(Q3) Is it likely that subjects received an unintended intervention (contamination or co-intervention) that may influence the results?**

4 Yes. As intended the ‘ CCMI sessions were longer than ACP sessions. Care managers demonstrated significantly higher global and behaviour ratings when conducting the CCMI session than ACP’. ‘ .. the care manager psychoeducation call on opioid risk probably functioned as an active intervention making it more difficult to discern differences between the two arms.’

5 No

6 Can’t tell

H) **ANALYSES**

**(Q1) Indicate the unit of allocation (circle one)**

community

organization/institution

practice/office

individual

**(Q2) Indicate the unit of analysis (circle one)**

community

organization/institution

practice/office

individual

**(Q3) Are the statistical methods appropriate for the study design?**

1 Yes

2 No

3 Can’t tell

**(Q4) Is the analysis performed by intervention allocation status (i.e. intention to treat) rather than the actual intervention received?**

1 Yes - all the randomised participants were included in the analysis.

2 No

3 Can’t tell

**GLOBAL RATING**

**COMPONENT RATINGS**

Please transcribe the information from the gray boxes on pages 1-4 onto this page. See the dictionary on how to rate this section.

| A  Selection Bias | STRONG  1 | MODERATE  2 | WEAK  3 |  |
| --- | --- | --- | --- | --- |
| B  Study Design | **STRONG**  **1** | **MODERATE**  **2** | **WEAK**  **3** |  |
| C  Confounders | **STRONG**  **1** | **MODERATE**  **2** | **WEAK**  **3** |  |
| D  Blinding | **STRONG**  **1** | **MODERATE**  **2** | **WEAK**  **3** |  |
| D  Data Collecting Method | **STRONG**  **1** | **MODERATE**  **2** | **WEAK**  **3** |  |
| F  Withdrawals and Dropouts | **STRONG**  **1** | **MODERATE**  **2** | **WEAK**  **3** | Not applicable |

**GLOBAL RATING FOR THIS PAPER** (circle one):

1 STRONG (no WEAK ratings)

2 MODERATE (one WEAK rating)

3 WEAK (two or more WEAK ratings)

With both reviewers discussing the ratings:

Is there a discrepancy between the two reviewers with component (A-F) ratings?

Yes

No

If yes, Indicate the reason for the discrepancy

1 Oversight

2 Differences in interpretation of criteria

3 Differences in interpretation of study

Final Decision of both reviewers:

**1 STRONG**

**2 MODERATE**

**3 WEAK**

Studies Quality - Risk of Bias

**The Effective Public Health Practice Project (EPHPP) checklist criteria for each study**

# STUDY: Bushey et al., 2022

**COMPONENT RATINGS**

A) **Selection of Bias**

**(Q1) Are the individuals selected to participate in the study likely to be representative of the target population?**

1 Very likely

2 Somewhat likely - potential patients screened from the medical database and contacted

3 Not likely

4 Can’t tell

**(Q2) What percentage of selected individuals agreed to participate?**

1 80 - 100% agreement

2 60 – 79% agreement

3 less than 60% agreement

4 Not applicable

5 Can’t tell

**RATE THIS SECTION**

**STRONG 1**

**MODERATE 2**

**WEAK 3**

B) **STUDY DESIGN**

Indicate the study design

1 Randomized controlled trial

2 Controlled clinical trial

3 Cohort analytic (two group pre + post)

4 Case-control

5 Cohort (one group pre + post (before and after))

6 Interrupted time series

7 Other specify ____________________________

8 Can’t tell

Was the study described as randomized? If NO, go to Component C.

No Yes

If Yes, was the method of randomization described? (See dictionary)

No Yes

If Yes, was the method appropriate?

YES ‘ a master’s level study statistician used a random-number table to generate the allocation sequence, stratified by study side. We did not stratify by history of depression or substance use disorder because we expect randomisation to balance these potential cofounders.’

**RATE THIS SECTION**

**STRONG 1**

**MODERATE 2**

**WEAK 3**

C) **CONFOUNDERS**

**(Q1) Were there important differences between groups prior to the intervention?**

1 Yes

2 No

3 Can’t tell

The following are examples of confounders:

1 Race

2 Sex

3 Marital status/family

4 Age

5 SES (income or class)

6 Education

7 Health status

8 Pre-intervention score on outcome measure

(**Q2) If yes, indicate the percentage of relevant confounders that were controlled (either in the design (e.g. stratification, matching) or analysis)?**

1 80 – 100% (most)

2 60 – 79% (some)

3 Less than 60% (few or none)

4 Can’t Tell

**RATE THIS SECTION**

**STRONG 1**

**MODERATE 2**

**WEAK 3**

D) **BLINDING**

**(Q1) Was (were) the outcome assessor(s) aware of the intervention or exposure status of participants?**

1 Yes

2 No

3 Can’t tell

**(Q2) Were the study participants aware of the research question?**

1 Yes

2 No

3 Can’t tell

**RATE THIS SECTION**

**STRONG 1 MODERATE 2 WEAK 3**

E) **DATA COLLECTION METHODS**

**(Q1) Were data collection tools shown to be valid?**

1 Yes

2 No

3 Can’t tell

**(Q2) Were data collection tools shown to be reliable?**

1 Yes – yes BPI, COMM, RMDQ and PHQ-9 have known reliability and validity as measures for this population

2 No

3 Can’t tell

**RATE THIS SECTION**

**STRONG 1 MODERATE 2 WEAK 3**

F) **WITHDRAWALS AND DROP-OUTS**

**(Q1) Were withdrawals and drop-outs reported in terms of numbers and/or reasons per group?**

1 Yes – 29 (11.1%) withdrew from the study (12 in the MED na d17 in the CBT group)

2 No

3 Can’t tell

4 Not Applicable (i.e. one time surveys or interviews)

**(Q2) Indicate the percentage of participants completing the study. (If the percentage differs by groups, record the lowest).**

1 80 -100% - 240 (92.0%) completed assessments at 3months.

2 60 - 79%

3 less than 60%

4 Can’t tell

5 Not Applicable (i.e. Retrospective case-control)

**RATE THIS SECTION**

**STRONG 1 MODERATE 2 WEAK 3	Not Applicable**

G) **INTERVENTION INTEGRITY**

**(Q1) What percentage of participants received the allocated intervention or exposure of interest?**

1 80 -100% - in the MED group 90.8% attended at least 5 sessions.

2 60 - 79%

3 less than 60%

4 Can’t tell

**(Q2) Was the consistency of the intervention measured?**

1 Yes

2 No

3 Can’t tell

**(Q3) Is it likely that subjects received an unintended intervention (contamination or co-intervention) that may influence the results?**

4 Yes

5 No

6 Can’t tell

H) **ANALYSES**

**(Q1) Indicate the unit of allocation (circle one)**

community

organization/institution

practice/office

individual

**(Q2) Indicate the unit of analysis (circle one)**

community

organization/institution

practice/office

individual

**(Q3) Are the statistical methods appropriate for the study design?**

1 Yes

2 No

3 Can’t tell

**(Q4) Is the analysis performed by intervention allocation status (i.e. intention to treat) rather than the actual intervention received?**

1 Yes intention to treat in all randomised participants

2 No

3 Can’t tell

**GLOBAL RATING**

**COMPONENT RATINGS**

Please transcribe the information from the grey boxes on pages 1-4 onto this page. See the dictionary for how to rate this section.

| A  Selection Bias | STRONG  1 | MODERATE  2 | WEAK  3 |  |
| --- | --- | --- | --- | --- |
| B  Study Design | **STRONG**  **1** | **MODERATE**  **2** | **WEAK**  **3** |  |
| C  Confounders | **STRONG**  **1** | **MODERATE**  **2** | **WEAK**  **3** |  |
| D  Blinding | **STRONG**  **1** | **MODERATE**  **2** | **WEAK**  **3** |  |
| D  Data Collecting Method | **STRONG**  **1** | **MODERATE**  **2** | **WEAK**  **3** |  |
| F  Withdrawals and Dropouts | **STRONG**  **1** | **MODERATE**  **2** | **WEAK**  **3** | Not applicable |

**GLOBAL RATING FOR THIS PAPER** (circle one):

1 STRONG (no WEAK ratings)

2 MODERATE (one WEAK rating)

3 WEAK (two or more WEAK ratings)

With both reviewers discussing the ratings:

Is there a discrepancy between the two reviewers with component (A-F) ratings?

Yes

No

If yes, Indicate the reason for the discrepancy

1 Oversight

2 Differences in interpretation of criteria

3 Differences in interpretation of study

Final Decision of both reviewers:

**1 STRONG**

**2 MODERATE
3 WEAK**

Studies Quality - Risk of Bias

**The Effective Public Health Practice Project (EPHPP) checklist criteria for each study**

# STUDY: Darnall et al., 2018

**COMPONENT RATINGS**

A) **Selection of Bias**

**(Q1) Are the individuals selected to participate in the study likely to be representative of the target population?**

1 Very likely

2 Somewhat likely

3 Not likely voluntary – not much information provided

4 Can’t tell

**(Q2) What percentage of selected individuals agreed to participate?**

1 80 - 100% agreement

2 60 – 79% agreement from the 110 eligible patients 75% agreed to participate

3 less than 60% agreement

4 Not applicable

5 Can’t tell

**RATE THIS SECTION**

**STRONG 1**

**MODERATE 2**

**WEAK 3**

B) **STUDY DESIGN**

Indicate the study design

1 Randomized controlled trial

2 Controlled clinical trial

3 Cohort analytic (two group pre + post)

4 Case-control

5 Cohort (one group pre + post (before and after))

6 Interrupted time series

7 Other specify ____________________________

8 Can’t tell

Was the study described as randomized? If NO, go to Component C.

No Yes

If Yes, was the method of randomization described? (See dictionary)

No Yes

If Yes, was the method appropriate?

**RATE THIS SECTION**

**STRONG 1 MODERATE 2 WEAK 3**

C) **CONFOUNDERS**

**(Q1) Were there important differences between groups prior to the intervention?**

1 Yes

2 No

3 Can’t tell

The following are examples of confounders:

1 Race

2 Sex

3 Marital status/family

4 Age

5 SES (income or class)

6 Education

7 Health status

8 Pre-intervention score on outcome measure

(**Q2) If yes, indicate the percentage of relevant confounders that were controlled (either in the design (e.g. stratification, matching) or analysis)?**

1 80 – 100% (most)

2 60 – 79% (some)

3 Less than 60% (few or none)

4 Can’t Tell

**RATE THIS SECTION**

**STRONG 1 MODERATE 2 WEAK 3 Not applicable as there are no groups.**

D) **BLINDING**

**(Q1) Was (were) the outcome assessor(s) aware of the intervention or exposure status of participants?**

1 Yes

2 No

3 Can’t tell

**(Q2) Were the study participants aware of the research question?**

1 Yes

2 No

3 Can’t tell

**RATE THIS SECTION**

**STRONG 1 MODERATE 2 WEAK 3**

E) **DATA COLLECTION METHODS**

**(Q1) Were data collection tools shown to be valid?**

1 Yes

2 No

3 Can’t tell

**(Q2) Were data collection tools shown to be reliable?**

1 Yes

2 No

3 Can’t tell

**RATE THIS SECTION**

**STRONG 1 MODERATE 2 WEAK 3**

F) **WITHDRAWALS AND DROP-OUTS**

**(Q1) Were withdrawals and drop-outs reported in terms of numbers and/or reasons per group?**

1 Yes

2 No

3 Can’t tell

4 Not Applicable (i.e. one time surveys or interviews)

**(Q2) Indicate the percentage of participants completing the study. (If the percentage differs by groups, record the lowest).**

1 80 -100%

2 60 - 79%

3 less than 60%

4 Can’t tell

5 Not Applicable (i.e. Retrospective case-control)

**RATE THIS SECTION**

**STRONG 1 MODERATE 2 WEAK 3	Not Applicable**

G) **INTERVENTION INTEGRITY**

**(Q1) What percentage of participants received the allocated intervention or exposure of interest?**

1 80 -100%

2 60 - 79%

3 less than 60%

4 Can’t tell

**(Q2) Was the consistency of the intervention measured?**

1 Yes

2 No

3 Can’t tell

**(Q3) Is it likely that subjects received an unintended intervention (contamination or co-intervention) that may influence the results?**

4 Yes

5 No

6 Can’t tell

H) **ANALYSES**

**(Q1) Indicate the unit of allocation (circle one)**

community

organization/institution

practice/office

individual

**(Q2) Indicate the unit of analysis (circle one)**

community

organization/institution

practice/office

individual

**(Q3) Are the statistical methods appropriate for the study design?**

1 Yes

2 No – only completers were included in the analysis

3 Can’t tell

**(Q4) Is the analysis performed by intervention allocation status (i.e. intention to treat) rather than the actual intervention received?**

1 Yes

2 No - only completers were included in the analysis

3 Can’t tell

**GLOBAL RATING**

**COMPONENT RATINGS**

Please transcribe the information from the gray boxes on pages 1-4 onto this page. See dictionary on how to rate this section.

| A  Selection Bias | STRONG  1 | MODERATE  2 | WEAK  3 |  |
| --- | --- | --- | --- | --- |
| B  Study Design | **STRONG**  **1** | **MODERATE**  **2** | **WEAK**  **3** |  |
| C  Confounders | **STRONG**  **1** | **MODERATE**  **2** | **WEAK**  **3** | Not applicable |
| D  Blinding | **STRONG**  **1** | **MODERATE**  **2** | **WEAK**  **3** |  |
| D  Data Collecting Method | **STRONG**  **1** | **MODERATE**  **2** | **WEAK**  **3** |  |
| F  Withdrawals and Dropouts | **STRONG**  **1** | **MODERATE**  **2** | **WEAK**  **3** | Not applicable |

**GLOBAL RATING FOR THIS PAPER** (circle one):

1 STRONG (no WEAK ratings)

2 MODERATE (one WEAK rating)

3 WEAK (two or more WEAK ratings)

With both reviewers discussing the ratings:

Is there a discrepancy between the two reviewers with component (A-F) ratings?

Yes

No

If yes, Indicate the reason for the discrepancy

1 Oversight

2 Differences in interpretation of criteria

3 Differences in interpretation of study

Final Decision of both reviewers:

**1 STRONG**

**2 MODERATE
3 WEAK**

Studies Quality - Risk of Bias

**The Effective Public Health Practice Project (EPHPP) checklist criteria for each study**

# STUDY: DeBar et al., 2022

**COMPONENT RATINGS**

A) **SELECTION of BIAS**

**(Q1) Are the individuals selected to participate in the study likely to be representative of the target population?**

1 Very likely

2 Somewhat likely

3 Not likely

4 Can’t tell

**(Q2) What percentage of selected individuals agreed to participate?**

1 80 - 100% agreement

2 60 – 79% agreement

3 less than 60% agreement

4 Not applicable

5 Can’t tell

**RATE THIS SECTION**

**STRONG 1**

**MODERATE 2**

**WEAK 3**

B) **STUDY DESIGN**

Indicate the study design

1 Randomized controlled trial

2 Controlled clinical trial

3 Cohort analytic (two group pre + post)

4 Case-control

5 Cohort (one group pre + post (before and after))

6 Interrupted time series

7 Other specify ____________________________

8 Can’t tell

Was the study described as randomized? If NO, go to Component C.

No Yes

If Yes, was the method of randomization described? (See dictionary)

No Yes

If Yes, was the method appropriate?

**RATE THIS SECTION**

**STRONG 1 MODERATE 2 WEAK 3**

C) **CONFOUNDERS**

**(Q1) Were there important differences between groups prior to the intervention?**

1 Yes

2 No

3 Can’t tell

The following are examples of confounders:

1 Race

2 Sex

3 Marital status/family

4 Age

5 SES (income or class)

6 Education

7 Health status

8 Pre-intervention score on outcome measure

(**Q2) If yes, indicate the percentage of relevant confounders that were controlled (either in the design (e.g. stratification, matching) or analysis)?**

1 80 – 100% (most)

2 60 – 79% (some)

3 Less than 60% (few or none)

4 Can’t Tell

**RATE THIS SECTION**

**STRONG 1 MODERATE 2 WEAK 3**

D) **BLINDING**

**(Q1) Was (were) the outcome assessor(s) aware of the intervention or exposure status of participants?**

1 Yes

2 No

3 Can’t tell

**(Q2) Were the study participants aware of the research question?**

1 Yes

2 No

3 Can’t tell

**RATE THIS SECTION**

**STRONG 1**

**MODERATE 2**

**WEAK 3**

E) **DATA COLLECTION METHODS**

**(Q1) Were data collection tools shown to be valid?**

1 Yes BPI and RMDQ

2 No

3 Can’t tell

**(Q2) Were data collection tools shown to be reliable?**

1 Yes

2 No

3 Can’t tell

**RATE THIS SECTION**

**STRONG 1 MODERATE 2 WEAK 3**

F) **WITHDRAWALS AND DROP-OUTS**

**(Q1) Were withdrawals and drop-outs reported in terms of numbers and/or reasons per group?**

1 Yes

2 No

3 Can’t tell

4 Not Applicable (i.e. one time surveys or interviews)

**(Q2) Indicate the percentage of participants completing the study. (If the percentage differs by groups, record the lowest).**

1 80 -100%

2 60 - 79%

3 less than 60%

4 Can’t tell

5 Not Applicable (i.e. Retrospective case-control)

**RATE THIS SECTION**

**STRONG 1**

**MODERATE 2**

**WEAK 3**

**Not Applicable**

G) **INTERVENTION INTEGRITY**

**(Q1) What percentage of participants received the allocated intervention or exposure of interest?**

1 80 -100%

2 60 - 79% -‘ Those randomly assigned to the CBT intervention completed an average of 2.9 of 4 possible in-person sessions and 6.2 of 12 possible group sessions. We categorized 63% of intervention participants as “intervention completers” (that is, finished ≥2 in-person sessions and ≥6 group sessions).’

3 less than 60%

4 Can’t tell

**(Q2) Was the consistency of the intervention measured?**

1 Yes - ‘ Therapist adherence to the protocol was high (mean, 4.4 on a scale of 0 to 5) but with a fair degree of variability (SD, 0.51; range, 3 to 5).’

2 No

3 Can’t tell

**(Q3) Is it likely that subjects received an unintended intervention (contamination or co-intervention) that may influence the results?**

4 Yes

5 No

6 Can’t tell

H) **ANALYSES**

**(Q1) Indicate the unit of allocation (circle one)**

community

organization/institution

practice/office

individual

**(Q2) Indicate the unit of analysis (circle one)**

community

organization/institution

practice/office

individual

**(Q3) Are the statistical methods appropriate for the study design?**

1 Yes

2 No

3 Can’t tell

**(Q4) Is the analysis performed by intervention allocation status (i.e. intention to treat) rather than the actual intervention received?**

1 Yes

2 No

3 Can’t tell

**GLOBAL RATING**

**COMPONENT RATINGS**

Please transcribe the information from the grey boxes on pages 1-4 onto this page. See the dictionary on how to rate this section.

| A  Selection Bias | STRONG  1 | MODERATE  2 | WEAK  3 |  |
| --- | --- | --- | --- | --- |
| B  Study Design | **STRONG**  **1** | **MODERATE**  **2** | **WEAK**  **3** |  |
| C  Confounders | **STRONG**  **1** | **MODERATE**  **2** | **WEAK**  **3** |  |
| D  Blinding | **STRONG**  **1** | **MODERATE**  **2** | **WEAK**  **3** |  |
| D  Data Collecting Method | **STRONG**  **1** | **MODERATE**  **2** | **WEAK**  **3** |  |
| F  Withdrawals and Dropouts | **STRONG**  **1** | **MODERATE**  **2** | **WEAK**  **3** | Not applicable |

**GLOBAL RATING FOR THIS PAPER** (circle one):

1 STRONG (no WEAK ratings)

2 MODERATE (one WEAK rating)

3 WEAK (two or more WEAK ratings)

With both reviewers discussing the ratings:

Is there a discrepancy between the two reviewers with component (A-F) ratings?

Yes

No

If yes, Indicate the reason for the discrepancy

1 Oversight

2 Differences in interpretation of criteria

3 Differences in interpretation of study

Final Decision of both reviewers:

**1 STRONG**

**2 MODERATE
3 WEAK**

Studies Quality - Risk of Bias

**The Effective Public Health Practice Project (EPHPP) checklist criteria for each study**

STUDY: Garland et al., 2020 **( some information accessed on the Garland et al., 2019)**

**COMPONENT RATINGS**

A) **SELECTION OF BIAS**

**(Q1) Are the individuals selected to participate in the study likely to be representative of the target population?**

1 Very likely

2 Somewhat likely

3 Not likely – participants were recruited from medical records, letters, flyers and radio advertisements.

4 Can’t tell

**(Q2) What percentage of selected individuals agreed to participate?**

1 80 - 100% agreement

2 60 – 79% agreement – 126 eligible and 95 agreed to be randomised.

3 less than 60% agreement

4 Not applicable

5 Can’t tell

**RATE THIS SECTION**

**STRONG 1**

**MODERATE 2**

**WEAK 3**

B) **STUDY DESIGN**

Indicate the study design

1 Randomized controlled trial - computerized random number table was generated by a researcher who was uninvolved in assessment

2 Controlled clinical trial

3 Cohort analytic (two group pre + post)

4 Case-control

5 Cohort (one group pre + post (before and after))

6 Interrupted time series

7 Other specify ____________________________

8 Can’t tell

Was the study described as randomized? If NO, go to Component C.

No Yes

If Yes, was the method of randomization described? (See dictionary)

No Yes

If Yes, was the method appropriate? Yes ‘ computerized random number table was generated by a researcher who was uninvolved in assessment, treatment, or enrollment using simple randomization in blocks of varying sizes (2 – 4) to preserve unpredictability of allocation. The allocation list was stored in a protected file inaccessible to project staff involved in assessment or treatment. Before each assessment, participants were reminded to not disclose their group assignment to study staff. Following pre-intervention assessment, participants were randomly allocated by a project staff member who was uninvolved with assessment or treatment to one of two undisclosed treatment groups. To prevent bias and maintain allocation concealment, the identity of treatment allocation was not revealed to the participant until the time when he or she arrived to the first treatment session’

**RATE THIS SECTION**

**STRONG 1 MODERATE 2 WEAK 3**

C) **CONFOUNDERS**

**(Q1) Were there important differences between groups prior to the intervention?**

1 Yes ‘ Due to random chance, between-group differences in gender approached significance; thus, we explored gender differences in pre-treatment variables of interest. No significant differences were found, except for savoring, for which women reported higher values than men (p = .04).’

2 No

3 Can’t tell

The following are examples of confounders:

1 Race

2 Sex

3 Marital status/family

4 Age

5 SES (income or class)

6 Education

7 Health status

8 Pre-intervention score on outcome measure

(**Q2) If yes, indicate the percentage of relevant confounders that were controlled (either in the design (e.g. stratification, matching) or analysis)?**

1 80 – 100% (most) ?? ‘ Although the observed between-group differences in gender, income, education, pain duration, and opioid use durations were nonsignificant, to ensure the robustness of our findings, we elected to perform a second series of analyses that controlled for these variables as covariates. These analyses yielded nearly identical results, indicating a significant effect of MORE on the following variables of interest: positive affect (β = .31, p = .01), meaning in life (β = .41, p = .001), and self-transcendence (β = .56, p <.001), residualized change in pain at post-treatment (β = −.29, p = .01) and change in opioid misuse behaviors at 3-month follow-up (β = −.36, p = .009); savoring being the only exception, which approached significance (β = .22, p = .07). ‘

2 60 – 79% (some)

3 Less than 60% (few or none)

4 Can’t Tell

**RATE THIS SECTION**

**STRONG 1 MODERATE 2 WEAK 3**

D) **BLINDING**

**(Q1) Was (were) the outcome assessor(s) aware of the intervention or exposure status of participants?**

1 Yes

2 No

3 Can’t tell

**(Q2) Were the study participants aware of the research question?**

1 Yes

2 No

3 Can’t tell

**RATE THIS SECTION**

**STRONG 1 MODERATE 2 WEAK 3**

E) **DATA COLLECTION METHODS**

**(Q1) Were data collection tools shown to be valid?**

1 Yes

2 No

3 Can’t tell

**(Q2) Were data collection tools shown to be reliable?**

1 Yes

2 No

3 Can’t tell

**RATE THIS SECTION**

**STRONG 1 MODERATE 2 WEAK 3**

F) **WITHDRAWALS AND DROP-OUTS**

**(Q1) Were withdrawals and drop-outs reported in terms of numbers and/or reasons per group?**

1 Yes

2 No

3 Can’t tell

4 Not Applicable (i.e. one time surveys or interviews)

**(Q2) Indicate the percentage of participants completing the study. (If the percentage differs by groups, record the lowest).**

1 80 -100%

2 60 - 79%

3 less than 60% - 74% completers (attending 4 or more sessions in the MORE group). Although follow-up rate in the MORE group was 22/50 (40%) and in the SG group was 28/45 (62%)

4 Can’t tell

5 Not Applicable (i.e. Retrospective case-control)

**RATE THIS SECTION**

**STRONG 1 MODERATE 2 WEAK 3	Not Applicable**

G) **INTERVENTION INTEGRITY**

**(Q1) What percentage of participants received the allocated intervention or exposure of interest?**

1 80 -100%

2 60 - 79% - 74% of the MORE group completed 4 or more sessions

3 less than 60%

4 Can’t tell

**(Q2) Was the consistency of the intervention measured?**

1 Yes

2 No

3 Can’t tell

**(Q3) Is it likely that subjects received an unintended intervention (contamination or co-intervention) that may influence the results?**

4 Yes

5 No

6 Can’t tell

H) **ANALYSES**

**(Q1) Indicate the unit of allocation (circle one)**

community

organization/institution

practice/office

individual

**(Q2) Indicate the unit of analysis (circle one)**

community

organization/institution

practice/office

individual

**(Q3) Are the statistical methods appropriate for the study design?**

1 Yes

2 No

3 Can’t tell

**(Q4) Is the analysis performed by intervention allocation status (i.e. intention to treat) rather than the actual intervention received?**

1 Yes

2 No

3 Can’t tell

**GLOBAL RATING**

**COMPONENT RATINGS**

Please transcribe the information from the gray boxes on pages 1-4 onto this page. See dictionary on how to rate this section.

| A  Selection Bias | STRONG  1 | MODERATE  2 | WEAK  3 |  |
| --- | --- | --- | --- | --- |
| B  Study Design | **STRONG**  **1** | **MODERATE**  **2** | **WEAK**  **3** |  |
| C  Confounders | **STRONG**  **1** | **MODERATE**  **2** | **WEAK**  **3** |  |
| D  Blinding | **STRONG**  **1** | **MODERATE**  **2** | **WEAK**  **3** |  |
| E  Data Collecting Method | **STRONG**  **1** | **MODERATE**  **2** | **WEAK**  **3** |  |
| F  Withdrawals and Dropouts | **STRONG**  **1** | **MODERATE**  **2** | **WEAK**  **3** | Not applicable |

**GLOBAL RATING FOR THIS PAPER** (circle one):

1 STRONG (no WEAK ratings)

2 MODERATE (one WEAK rating)

3 WEAK (two or more WEAK ratings)

With both reviewers discussing the ratings:

Is there a discrepancy between the two reviewers with component (A-F) ratings?

Yes

No

If yes, Indicate the reason for the discrepancy

1 Oversight

2 Differences in interpretation of criteria

3 Differences in interpretation of study

Final Decision of both reviewers:

**1 STRONG**

**2 MODERATE
3 WEAK**

Studies Quality - Risk of Bias

**The Effective Public Health Practice Project (EPHPP) checklist criteria for each study**

# STUDY: Garland et al., 2022

**COMPONENT RATINGS**

A) **Selection of Bias**

**(Q1) Are the individuals selected to participate in the study likely to be representative of the target population?**

1 Very likely

2 Somewhat likely – Participants were recruited from electronic health record data, physician referrals, and community

advertisements.

3 Not likely

4 Can’t tell

**(Q2) What percentage of selected individuals agreed to participate?**

1 80 - 100% agreement

2 60 – 79% agreement

3 less than 60% agreement

4 Not applicable

5 Can’t tell

**RATE THIS SECTION**

**STRONG 1**

**MODERATE 2**

**WEAK 3**

B) **STUDY DESIGN**

Indicate the study design

1 Randomized controlled trial

2 Controlled clinical trial

3 Cohort analytic (two group pre + post)

4 Case-control

5 Cohort (one group pre + post (before and after))

6 Interrupted time series

7 Other specify ____________________________

8 Can’t tell

Was the study described as randomized? If NO, go to Component C.

No Yes

If Yes, was the method of randomization described? (See dictionary)

No Yes

If Yes, was the method appropriate?

**RATE THIS SECTION**

**STRONG 1 MODERATE 2 WEAK 3**

C) **CONFOUNDERS**

**(Q1) Were there important differences between groups prior to the intervention?**

1 Yes

2 No

3 Can’t tell

The following are examples of confounders:

1 Race

2 Sex

3 Marital status/family

4 Age

5 SES (income or class)

6 Education

7 Health status

8 Pre-intervention score on outcome measure

(**Q2) If yes, indicate the percentage of relevant confounders that were controlled (either in the design (e.g. stratification, matching) or analysis)?**

1 80 – 100% (most)

2 60 – 79% (some)

3 Less than 60% (few or none)

4 Can’t Tell

**RATE THIS SECTION**

**STRONG 1**

**MODERATE 2**

**WEAK 3**

D) **BLINDING**

**(Q1) Was (were) the outcome assessor(s) aware of the intervention or exposure status of participants?**

1 Yes

2 No

3 Can’t tell

**(Q2) Were the study participants aware of the research question?**

1 Yes

2 No

3 Can’t tell

**RATE THIS SECTION**

**STRONG 1**

**MODERATE 2**

**WEAK 3**

E) **DATA COLLECTION METHODS**

**(Q1) Were data collection tools shown to be valid?**

1 Yes

2 No

3 Can’t tell

**(Q2) Were data collection tools shown to be reliable?**

1 Yes

2 No

3 Can’t tell

**RATE THIS SECTION**

**STRONG 1 MODERATE 2 WEAK 3**

F) **WITHDRAWALS AND DROP-OUTS**

**(Q1) Were withdrawals and drop-outs reported in terms of numbers and/or reasons per group?**

1 Yes

2 No

3 Can’t tell

4 Not Applicable (i.e. one time surveys or interviews)

**(Q2) Indicate the percentage of participants completing the study. (If the percentage differs by groups, record the lowest).**

1 80 -100% - ‘ A total of 203 participants (81.2%) completed treatment.

2 60 - 79%

3 less than 60%

4 Can’t tell

5 Not Applicable (i.e. Retrospective case-control)

**RATE THIS SECTION**

**STRONG 1 MODERATE 2 WEAK 3	Not Applicable**

G) **INTERVENTION INTEGRITY**

**(Q1) What percentage of participants received the allocated intervention or exposure of interest?**

1 80 -100%

2 60 - 79%

3 less than 60%

4 Can’t tell

**(Q2) Was the consistency of the intervention measured?**

1 Yes ‘ Sessions were audio recorded, and treatment fidelity was monitored using validated measures.’

2 No

3 Can’t tell

**(Q3) Is it likely that subjects received an unintended intervention (contamination or co-intervention) that may influence the results?**

4 Yes

5 No ‘ therapist adherence and competence measures were excellent, indicating that therapists skilfully adhere to each of the standardized protocols with no treatment diffusion.’

6 Can’t tell

H) **ANALYSES**

**(Q1) Indicate the unit of allocation (circle one)**

community

organization/institution

practice/office

individual

**(Q2) Indicate the unit of analysis (circle one)**

community

organization/institution

practice/office

individual

**(Q3) Are the statistical methods appropriate for the study design?**

1 Yes

2 No

3 Can’t tell

**(Q4) Is the analysis performed by intervention allocation status (i.e. intention to treat) rather than the actual intervention received?**

1 Yes

2 No

3 Can’t tell

**GLOBAL RATING**

**COMPONENT RATINGS**

Please transcribe the information from the grey boxes on pages 1-4 onto this page. See the dictionary on how to rate this section.

| A  Selection Bias | STRONG  1 | MODERATE  2 | WEAK  3 |  |
| --- | --- | --- | --- | --- |
| B  Study Design | **STRONG**  **1** | **MODERATE**  **2** | **WEAK**  **3** |  |
| C  Confounders | **STRONG**  **1** | **MODERATE**  **2** | **WEAK**  **3** |  |
| D  Blinding | **STRONG**  **1** | **MODERATE**  **2** | **WEAK**  **3** |  |
| D  Data Collecting Method | **STRONG**  **1** | **MODERATE**  **2** | **WEAK**  **3** |  |
| F  Withdrawals and Dropouts | **STRONG**  **1** | **MODERATE**  **2** | **WEAK**  **3** | Not applicable |

**GLOBAL RATING FOR THIS PAPER** (circle one):

1 STRONG (no WEAK ratings)

2 MODERATE (one WEAK rating)

3 WEAK (two or more WEAK ratings)

With both reviewers discussing the ratings:

Is there a discrepancy between the two reviewers with component (A-F) ratings?

Yes

No

If yes, Indicate the reason for the discrepancy

1 Oversight

2 Differences in interpretation of criteria

3 Differences in interpretation of study

Final Decision of both reviewers:

**1 STRONG**

**2 MODERATE
3 WEAK**

Studies Quality - Risk of Bias

**The Effective Public Health Practice Project (EPHPP) checklist criteria for each study**

# STUDY: Mehl-Madrona et al., 2016

**COMPONENT RATINGS**

A) **SELECTION of BIAS**

**(Q1) Are the individuals selected to participate in the study likely to be representative of the target population?**

1 Very likely

2 Somewhat likely – referred from clinic

3 Not likely

4 Can’t tell

**(Q2) What percentage of selected individuals agreed to participate?**

1 80 - 100% agreement

2 60 – 79% agreement – ‘from a total of 207 patients seeking pain services , 84 agreed to participate.’

3 less than 60% agreement

4 Not applicable

5 Can’t tell

**RATE THIS SECTION**

**STRONG 1**

**MODERATE 2**

**WEAK 3**

B) **STUDY DESIGN**

Indicate the study design

1 Randomized controlled trial

2 Controlled clinical trial

3 Cohort analytic (two group pre + post) – intervention arm comparing to a matching cohort, through the use of electronic health record a matched comparison population from the same practice was generated

4 Case-control

5 Cohort (one group pre + post (before and after))

6 Interrupted time series

7 Other specify ____________________________

8 Can’t tell

Was the study described as randomized? If NO, go to Component C.

No Yes

If Yes, was the method of randomization described? (See dictionary)

No Yes

If Yes, was the method appropriate?

**RATE THIS SECTION**

**STRONG 1 MODERATE 2 WEAK 3**

C) **CONFOUNDERS**

**(Q1) Were there important differences between groups prior to the intervention?**

1 Yes

2 No – a matching cohort was created using the electronic health system of the GP practice , matching was done using age, sex, dose of morphine equivalents.

3 Can’t tell

The following are examples of confounders:

1 Race

2 Sex

3 Marital status/family

4 Age

5 SES (income or class)

6 Education

7 Health status

8 Pre-intervention score on outcome measure

(**Q2) If yes, indicate the percentage of relevant confounders that were controlled (either in the design (e.g. stratification, matching) or analysis)?**

1 80 – 100% (most)

2 60 – 79% (some)

3 Less than 60% (few or none)

4 Can’t Tell

**RATE THIS SECTION**

**STRONG 1 MODERATE 2 WEAK 3**

D) **BLINDING**

**(Q1) Was (were) the outcome assessor(s) aware of the intervention or exposure status of participants?**

1 Yes

2 No

3 Can’t tell

**(Q2) Were the study participants aware of the research question?**

1 Yes

2 No

3 Can’t tell

**RATE THIS SECTION**

**STRONG 1 MODERATE 2 WEAK 3**

E) **DATA COLLECTION METHODS**

**(Q1) Were data collection tools shown to be valid?**

1 Yes used change in opioid dosage in morphine equivalents from electronic health records and My medical Outcome profile questionnaire.

2 No

3 Can’t tell

**(Q2) Were data collection tools shown to be reliable?**

1 Yes

2 No

3 Can’t tell

**RATE THIS SECTION**

**STRONG 1 MODERATE 2 WEAK 3**

F) **WITHDRAWALS AND DROP-OUTS**

**(Q1) Were withdrawals and drop-outs reported in terms of numbers and/or reasons per group?**

1 Yes

2 No

3 Can’t tell

4 Not Applicable (i.e. one time surveys or interviews)

**(Q2) Indicate the percentage of participants completing the study. (If the percentage differs by groups, record the lowest).**

1 80 -100%

2 60 - 79%

3 less than 60% from the 84 enrolled, 41 did not reach 6 months of attending group medical visits.

4 Can’t tell

5 Not Applicable (i.e. Retrospective case-control)

**RATE THIS SECTION**

**STRONG 1**

**MODERATE 2**

**WEAK 3**

**Not Applicable**

G) **INTERVENTION INTEGRITY**

**(Q1) What percentage of participants received the allocated intervention or exposure of interest?**

1 80 -100%

2 60 - 79%

3 less than 60%

4 Can’t tell

**(Q2) Was the consistency of the intervention measured?**

1 Yes

2 No

3 Can’t tell

**(Q3) Is it likely that subjects received an unintended intervention (contamination or co-intervention) that may influence the results?**

4 Yes

5 No

6 Can’t tell

H) **ANALYSES**

**(Q1) Indicate the unit of allocation (circle one)**

community

organization/institution

practice/office

individual

**(Q2) Indicate the unit of analysis (circle one)**

community

organization/institution

practice/office

individual

**(Q3) Are the statistical methods appropriate for the study design?**

1 Yes

2 No

3 Can’t tell

**(Q4) Is the analysis performed by intervention allocation status (i.e. intention to treat) rather than the actual intervention received?**

1 Yes

2 No

3 Can’t tell

**GLOBAL RATING**

**COMPONENT RATINGS**

Please transcribe the information from the gray boxes on pages 1-4 onto this page. See dictionary on how to rate this section.

| A  Selection Bias | STRONG  1 | MODERATE  2 | WEAK  3 |  |
| --- | --- | --- | --- | --- |
| B  Study Design | **STRONG**  **1** | **MODERATE**  **2** | **WEAK**  **3** |  |
| C  Confounders | **STRONG**  **1** | **MODERATE**  **2** | **WEAK**  **3** |  |
| D  Blinding | **STRONG**  **1** | **MODERATE**  **2** | **WEAK**  **3** |  |
| D  Data Collecting Method | **STRONG**  **1** | **MODERATE**  **2** | **WEAK**  **3** |  |
| F  Withdrawals and Dropouts | **STRONG**  **1** | **MODERATE**  **2** | **WEAK**  **3** | Not applicable |

**GLOBAL RATING FOR THIS PAPER** (circle one):

1 STRONG (no WEAK ratings)

2 MODERATE (one WEAK rating)

3 WEAK (two or more WEAK ratings)

With both reviewers discussing the ratings:

Is there a discrepancy between the two reviewers with component (A-F) ratings?

Yes

No

If yes, Indicate the reason for the discrepancy

1 Oversight

2 Differences in interpretation of criteria

3 Differences in interpretation of study

Final Decision of both reviewers:

**1 STRONG**

**2 MODERATE
3 WEAK**

Studies Quality - Risk of Bias

**The Effective Public Health Practice Project (EPHPP) checklist criteria for each study**

# STUDY: Sandhu et al., 2023

**COMPONENT RATINGS**

A) **Selection of Bias**

**(Q1) Are the individuals selected to participate in the study likely to be representative of the target population?**

1 Very likely

2 Somewhat likely

3 Not likely

4 Can’t tell

**(Q2) What percentage of selected individuals agreed to participate?**

1 80 - 100% agreement

2 60 – 79% agreement

3 less than 60% agreement from 20900 patients approached, 608 recruited.

4 Not applicable

5 Can’t tell

**RATE THIS SECTION**

**STRONG 1**

**MODERATE 2**

**WEAK 3**

B) **STUDY DESIGN**

Indicate the study design

1 Randomized controlled trial

2 Controlled clinical trial

3 Cohort analytic (two group pre + post)

4 Case-control

5 Cohort (one group pre + post (before and after))

6 Interrupted time series

7 Other specify ____________________________

8 Can’t tell

Was the study described as randomized? If NO, go to Component C.

No Yes

If Yes, was the method of randomization described? (See dictionary)

No Yes ‘ Randomization was performed by the Warwick Clinical Trials Unit programming team using Structured Query Language.’

If Yes, was the method appropriate?

**RATE THIS SECTION**

**STRONG 1 MODERATE 2 WEAK 3**

C) **CONFOUNDERS**

**(Q1) Were there important differences between groups prior to the intervention?**

1 Yes

2 No it was stratified at randomisation based on geographic area, baseline score for pain, baseline morphine dose.

3 Can’t tell

The following are examples of confounders:

1 Race

2 Sex

3 Marital status/family

4 Age

5 SES (income or class)

6 Education

7 Health status

8 Pre-intervention score on outcome measure

(**Q2) If yes, indicate the percentage of relevant confounders that were controlled (either in the design (e.g. stratification, matching) or analysis)?**

1 80 – 100% (most)

2 60 – 79% (some)

3 Less than 60% (few or none)

4 Can’t Tell

**RATE THIS SECTION**

**STRONG 1 MODERATE 2 WEAK 3**

D) **BLINDING**

**(Q1) Was (were) the outcome assessor(s) aware of the intervention or exposure status of participants?**

1 Yes

2 No

3 Can’t tell

**(Q2) Were the study participants aware of the research question?**

1 Yes

2 No

3 Can’t tell

**RATE THIS SECTION**

**STRONG 1 MODERATE 2 WEAK 3**

E) **DATA COLLECTION METHODS**

**(Q1) Were data collection tools shown to be valid?**

1 Yes

2 No

3 Can’t tell

**(Q2) Were data collection tools shown to be reliable?**

1 Yes

2 No

3 Can’t tell

**RATE THIS SECTION**

**STRONG 1 MODERATE 2 WEAK 3**

F) **WITHDRAWALS AND DROP-OUTS**

**(Q1) Were withdrawals and drop-outs reported in terms of numbers and/or reasons per group?**

1 Yes

2 No

3 Can’t tell

4 Not Applicable (i.e. one time surveys or interviews)

**(Q2) Indicate the percentage of participants completing the study. (If the percentage differs by groups, record the lowest).**

1 80 -100%

2 60 - 79%

3 less than 60% ‘47% achieved full adherence to the program’

4 Can’t tell

5 Not Applicable (i.e. Retrospective case-control)

**RATE THIS SECTION**

**STRONG 1 MODERATE 2 WEAK 3	Not Applicable**

G) **INTERVENTION INTEGRITY**

**(Q1) What percentage of participants received the allocated intervention or exposure of interest?**

1 80 -100%

2 60 - 79%

3 less than 60%

4 Can’t tell

**(Q2) Was the consistency of the intervention measured?**

1 Yes

2 No

3 Can’t tell

**(Q3) Is it likely that subjects received an unintended intervention (contamination or co-intervention) that may influence the results?**

4 Yes

5 No

6 Can’t tell

H) **ANALYSES**

**(Q1) Indicate the unit of allocation (circle one)**

community

organization/institution

practice/office

individual

**(Q2) Indicate the unit of analysis (circle one)**

community

organization/institution

practice/office

individual

**(Q3) Are the statistical methods appropriate for the study design?**

1 Yes

2 No

3 Can’t tell

**(Q4) Is the analysis performed by intervention allocation status (i.e. intention to treat) rather than the actual intervention received?**

1 Yes

2 No

3 Can’t tell

**GLOBAL RATING**

**COMPONENT RATINGS**

Please transcribe the information from the gray boxes on pages 1-4 onto this page. See dictionary on how to rate this section.

| A  Selection Bias | STRONG  1 | MODERATE  2 | WEAK  3 |  |
| --- | --- | --- | --- | --- |
| B  Study Design | **STRONG**  **1** | **MODERATE**  **2** | **WEAK**  **3** |  |
| C  Confounders | **STRONG**  **1** | **MODERATE**  **2** | **WEAK**  **3** |  |
| D  Blinding | **STRONG**  **1** | **MODERATE**  **2** | **WEAK**  **3** |  |
| D  Data Collecting Method | **STRONG**  **1** | **MODERATE**  **2** | **WEAK**  **3** |  |
| F  Withdrawals and Dropouts | **STRONG**  **1** | **MODERATE**  **2** | **WEAK**  **3** | Not applicable |

**GLOBAL RATING FOR THIS PAPER** (circle one):

1 STRONG (no WEAK ratings)

2 MODERATE (one WEAK rating)

3 WEAK (two or more WEAK ratings)

With both reviewers discussing the ratings:

Is there a discrepancy between the two reviewers with component (A-F) ratings?

Yes

No

If yes, Indicate the reason for the discrepancy

1 Oversight

2 Differences in interpretation of criteria

3 Differences in interpretation of study

Final Decision of both reviewers:

**1 STRONG**

**2 MODERATE
3 WEAK**

Studies Quality - Risk of Bias

**The Effective Public Health Practice Project (EPHPP) checklist criteria for each study**

# STUDY: Scott et al., 2020

**COMPONENT RATINGS**

A) **Selection of Bias**

**(Q1) Are the individuals selected to participate in the study likely to be representative of the target population?**

1 Very likely

2 Somewhat likely – referred by clinic

3 Not likely

4 Can’t tell

**(Q2) What percentage of selected individuals agreed to participate?**

1 80 - 100% agreement

2 60 – 79% agreement

3 less than 60% agreement approached 59, enrolled 34.

4 Not applicable

5 Can’t tell

**RATE THIS SECTION**

**STRONG 1**

**MODERATE 2**

**WEAK 3**

B) **STUDY DESIGN**

Indicate the study design

1 Randomized controlled trial

2 Controlled clinical trial

3 Cohort analytic (two group pre + post)

4 Case-control

5 Cohort (one group pre + post (before and after))

6 Interrupted time series

7 Other specify ____________________________

8 Can’t tell

Was the study described as randomized? If NO, go to Component C.

No Yes

If Yes, was the method of randomization described? (See dictionary)

No Yes

If Yes, was the method appropriate?

**RATE THIS SECTION**

**STRONG 1 MODERATE 2 WEAK 3**

C) **CONFOUNDERS**

**(Q1) Were there important differences between groups prior to the intervention?**

1 Yes

2 No

3 Can’t tell

The following are examples of confounders:

1 Race

2 Sex

3 Marital status/family

4 Age

5 SES (income or class)

6 Education

7 Health status

8 Pre-intervention score on outcome measure

(**Q2) If yes, indicate the percentage of relevant confounders that were controlled (either in the design (e.g. stratification, matching) or analysis)?**

1 80 – 100% (most)

2 60 – 79% (some)

3 Less than 60% (few or none)

4 Can’t Tell

**RATE THIS SECTION**

**STRONG 1 MODERATE 2 WEAK 3Not applicable. One group**

D) **BLINDING**

**(Q1) Was (were) the outcome assessor(s) aware of the intervention or exposure status of participants?**

1 Yes

2 No

3 Can’t tell

**(Q2) Were the study participants aware of the research question?**

1 Yes

2 No

3 Can’t tell

**RATE THIS SECTION**

**STRONG 1 MODERATE 2 WEAK 3**

E) **DATA COLLECTION METHODS**

**(Q1) Were data collection tools shown to be valid?**

1 Yes

2 No

3 Can’t tell

**(Q2) Were data collection tools shown to be reliable?**

1 Yes

2 No

3 Can’t tell

**RATE THIS SECTION**

**STRONG 1 MODERATE 2 WEAK 3**

F) **WITHDRAWALS AND DROP-OUTS**

**(Q1) Were withdrawals and drop-outs reported in terms of numbers and/or reasons per group?**

1 Yes

2 No

3 Can’t tell

4 Not Applicable (i.e. one time surveys or interviews)

**(Q2) Indicate the percentage of participants completing the study. (If the percentage differs by groups, record the lowest).**

1 80 -100%

2 60 - 79%

3 less than 60%

4 Can’t tell

5 Not Applicable (i.e. Retrospective case-control)

**RATE THIS SECTION**

**STRONG 1 MODERATE 2 WEAK 3	Not Applicable**

G) **INTERVENTION INTEGRITY**

**(Q1) What percentage of participants received the allocated intervention or exposure of interest?**

1 80 -100%

2 60 - 79%

3 less than 60%

4 Can’t tell

**(Q2) Was the consistency of the intervention measured?**

1 Yes

2 No

3 Can’t tell

**(Q3) Is it likely that subjects received an unintended intervention (contamination or co-intervention) that may influence the results?**

4 Yes

5 No

6 Can’t tell

H) **ANALYSES**

**(Q1) Indicate the unit of allocation (circle one)**

community

organization/institution

practice/office

individual

**(Q2) Indicate the unit of analysis (circle one)**

community

organization/institution

practice/office

individual

**(Q3) Are the statistical methods appropriate for the study design?**

1 Yes

2 No

3 Can’t tell

**(Q4) Is the analysis performed by intervention allocation status (i.e. intention to treat) rather than the actual intervention received?**

1 Yes

2 No

3 Can’t tell

**GLOBAL RATING**

**COMPONENT RATINGS**

Please transcribe the information from the gray boxes on pages 1-4 onto this page. See dictionary on how to rate this section.

| A  Selection Bias | STRONG  1 | MODERATE  2 | WEAK  3 |  |
| --- | --- | --- | --- | --- |
| B  Study Design | **STRONG**  **1** | **MODERATE**  **2** | **WEAK**  **3** |  |
| C  Confounders | **STRONG**  **1** | **MODERATE**  **2** | **WEAK**  **3** | Not applicable |
| D  Blinding | **STRONG**  **1** | **MODERATE**  **2** | **WEAK**  **3** |  |
| D  Data Collecting Method | **STRONG**  **1** | **MODERATE**  **2** | **WEAK**  **3** |  |
| F  Withdrawals and Dropouts | **STRONG**  **1** | **MODERATE**  **2** | **WEAK**  **3** | Not applicable |

**GLOBAL RATING FOR THIS PAPER** (circle one):

1 STRONG (no WEAK ratings)

2 MODERATE (one WEAK rating)

3 WEAK (two or more WEAK ratings)

With both reviewers discussing the ratings:

Is there a discrepancy between the two reviewers with component (A-F) ratings?

Yes

No

If yes, Indicate the reason for the discrepancy

1 Oversight

2 Differences in interpretation of criteria

3 Differences in interpretation of study

Final Decision of both reviewers:

**1 STRONG**

**2 MODERATE
3 WEAK**

Studies Quality - Risk of Bias

**The Effective Public Health Practice Project (EPHPP) checklist criteria for each study**

# **STUDY: Seal et al., 2020**

**COMPONENT RATINGS**

A) **SELECTION OF BIAS**

**(Q1) Are the individuals selected to participate in the study likely to be representative of the target population?**

1 Very likely

2 Somewhat likely

3 Not likely

4 Can’t tell – prospective cohort design. The participants were matched sequentially and they were stratified. It does not specify if the sample was randomly selected.

**(Q2) What percentage of selected individuals agreed to participate?**

1 80 - 100% agreement

2 60 – 79% agreement

3 less than 60% agreement

4 Not applicable

5 Can’t tell

**RATE THIS SECTION**

**STRONG 1**

**MODERATE 2**

**WEAK 3**

B) **STUDY DESIGN**

Indicate the study design

1 Randomized controlled trial

2 Controlled clinical trial

3 Cohort analytic (two group pre + post)

4 Case-control

5 Cohort (one group pre + post (before and after))

6 Interrupted time series

7 Other specify ____________________________

8 Can’t tell

Was the study described as randomized? If NO, go to Component C.

No Yes

If Yes, was the method of randomization described? (See dictionary)

No Yes

If Yes, was the method appropriate?

**RATE THIS SECTION**

**STRONG 1**

**MODERATE 2**

**WEAK 3**

C) **CONFOUNDERS**

**(Q1) Were there important differences between groups prior to the intervention?**

1 Yes

2 No – ‘ Patients were matched on age, sex, psychiatric diagnoses, and baseline opioid dose.’

3 Can’t tell

The following are examples of confounders:

1 Race

2 Sex

3 Marital status/family

4 Age

5 SES (income or class)

6 Education

7 Health status

8 Pre-intervention score on outcome measure

(**Q2) If yes, indicate the percentage of relevant confounders that were controlled (either in the design (e.g. stratification, matching) or analysis)?**

1 80 – 100% (most)

2 60 – 79% (some)

3 Less than 60% (few or none)

4 Can’t Tell

**RATE THIS SECTION**

**STRONG 1 MODERATE 2 WEAK 3**

D) **BLINDING**

**(Q1) Was (were) the outcome assessor(s) aware of the intervention or exposure status of participants?**

1 Yes

2 No

3 Can’t tell – not applicable. Data extracted from the electronic health system. Participants did not complete assessments.

**(Q2) Were the study participants aware of the research question?**

1 Yes

2 No

3 Can’t tell Not applicable. Data extracted from the electronic health system. Participants did not complete assessments.

**RATE THIS SECTION**

**STRONG 1 MODERATE 2 WEAK 3 X not applicable**

E) **DATA COLLECTION METHODS**

**(Q1) Were data collection tools shown to be valid?**

1 Yes electronic health records

2 No

3 Can’t tell

**(Q2) Were data collection tools shown to be reliable?**

1 Yes electronic health records

2 No

3 Can’t tell

**RATE THIS SECTION**

**STRONG 1 MODERATE 2 WEAK 3**

F) **WITHDRAWALS AND DROP-OUTS**

**(Q1) Were withdrawals and drop-outs reported in terms of numbers and/or reasons per group?**

1 Yes

2 No

3 Can’t tell

4 Not Applicable (i.e. one time surveys or interviews)

**(Q2) Indicate the percentage of participants completing the study. (If the percentage differs by groups, record the lowest).**

1 80 -100%

2 60 - 79%

3 less than 60%

4 Can’t tell

5 Not Applicable (i.e. Retrospective case-control)

**RATE THIS SECTION**

**STRONG 1 MODERATE 2 WEAK 3	Not Applicable**

G) **INTERVENTION INTEGRITY**

**(Q1) What percentage of participants received the allocated intervention or exposure of interest?**

1 80 -100%

2 60 - 79%

3 less than 60%

4 Can’t tell

NA- quality improvement cohort study

**(Q2) Was the consistency of the intervention measured?**

1 Yes

2 No

3 Can’t tell

NA- quality improvement cohort study

**(Q3) Is it likely that subjects received an unintended intervention (contamination or co-intervention) that may influence the results?**

4 Yes

5 No

6 Can’t tell

NA- quality improvement cohort study

H) **ANALYSES**

**(Q1) Indicate the unit of allocation (circle one)**

community

organization/institution

practice/office

individual

**(Q2) Indicate the unit of analysis (circle one)**

community

organization/institution

practice/office

individual

**(Q3) Are the statistical methods appropriate for the study design?**

1 Yes

2 No

3 Can’t tell

**(Q4) Is the analysis performed by intervention allocation status (i.e. intention to treat) rather than the actual intervention received?**

1 Yes

2 No

3 Can’t tell

**GLOBAL RATING**

**COMPONENT RATINGS**

Please transcribe the information from the gray boxes on pages 1-4 onto this page. See dictionary on how to rate this section.

| A  Selection Bias | STRONG  1 | MODERATE  2 | WEAK  3 |  |
| --- | --- | --- | --- | --- |
| B  Study Design | **STRONG**  **1** | **MODERATE**  **2** | **WEAK**  **3** |  |
| C  Confounders | **STRONG**  **1** | **MODERATE**  **2** | **WEAK**  **3** |  |
| D  Blinding | **STRONG**  **1** | **MODERATE**  **2** | **WEAK**  **3** | N/A |
| D  Data Collecting Method | **STRONG**  **1** | **MODERATE**  **2** | **WEAK**  **3** |  |
| F  Withdrawals and Dropouts | **STRONG**  **1** | **MODERATE**  **2** | **WEAK**  **3** | Not applicable |

**GLOBAL RATING FOR THIS PAPER** (circle one):

1 STRONG (no WEAK ratings)

2 MODERATE (one WEAK rating)

3 WEAK (two or more WEAK ratings)

With both reviewers discussing the ratings:

Is there a discrepancy between the two reviewers with component (A-F) ratings?

Yes

No

If yes, Indicate the reason for the discrepancy

1 Oversight

2 Differences in interpretation of criteria

3 Differences in interpretation of study

Final Decision of both reviewers:

**1 STRONG**

**2 MODERATE
3 WEAK**

Studies Quality - Risk of Bias

**The Effective Public Health Practice Project (EPHPP) checklist criteria for each study**

# STUDY: Sullivan et al., 2017

**COMPONENT RATINGS**

A) **SELECTION OF BIAS**

**(Q1) Are the individuals selected to participate in the study likely to be representative of the target population?**

1 Very likely

2 Somewhat likely

3 Not likely - clinic referrals and self-referals

4 Can’t tell

**(Q2) What percentage of selected individuals agreed to participate?**

1 80 - 100% agreement

2 60 – 79% agreement

3 less than 60% agreement 76 eleigible and contacted and 35 participants

4 Not applicable

5 Can’t tell

**RATE THIS SECTION**

**STRONG 1**

**MODERATE 2**

**WEAK 3**

B) **STUDY DESIGN**

Indicate the study design

1 Randomized controlled trial

2 Controlled clinical trial

3 Cohort analytic (two group pre + post)

4 Case-control

5 Cohort (one group pre + post (before and after))

6 Interrupted time series

7 Other specify ____________________________

8 Can’t tell

Was the study described as randomized? If NO, go to Component C.

No Yes

If Yes, was the method of randomization described? (See dictionary)

No Yes

If Yes, was the method appropriate? Yes. ‘ participants were randomized 1:1 to receive either the opioid taper intervention or usual care according to a computer-generated randomization list in sealed envelopes.’

**RATE THIS SECTION**

**STRONG 1 MODERATE 2 WEAK 3**

C) **CONFOUNDERS**

**(Q1) Were there important differences between groups prior to the intervention?**

1 Yes

2 No - stratified randomisation

3 Can’t tell

The following are examples of confounders:

1 Race

2 Sex

3 Marital status/family

4 Age

5 SES (income or class)

6 Education

7 Health status

8 Pre-intervention score on outcome measure

(**Q2) If yes, indicate the percentage of relevant confounders that were controlled (either in the design (e.g. stratification, matching) or analysis)?**

1 80 – 100% (most)

2 60 – 79% (some)

3 Less than 60% (few or none)

4 Can’t Tell

**RATE THIS SECTION**

**STRONG 1 MODERATE 2 WEAK 3**

D) **BLINDING**

**(Q1) Was (were) the outcome assessor(s) aware of the intervention or exposure status of participants?**

1 Yes

2 No

3 Can’t tell – no information provided

**(Q2) Were the study participants aware of the research question?**

1 Yes

2 No

3 Can’t tell

**RATE THIS SECTION**

**STRONG 1 MODERATE 2 WEAK 3**

E) **DATA COLLECTION METHODS**

**(Q1) Were data collection tools shown to be valid?**

1 Yes self-reported and medical records

2 No

3 Can’t tell

**(Q2) Were data collection tools shown to be reliable?**

1 Yes

2 No

3 Can’t tell

**RATE THIS SECTION**

**STRONG 1 MODERATE 2 WEAK 3**

F) **WITHDRAWALS AND DROP-OUTS**

**(Q1) Were withdrawals and drop-outs reported in terms of numbers and/or reasons per group?**

1 Yes

2 No

3 Can’t tell

4 Not Applicable (i.e. one time surveys or interviews)

**(Q2) Indicate the percentage of participants completing the study. (If the percentage differs by groups, record the lowest).**

1 80 -100%

2 60 - 79%

3 less than 60%

4 Can’t tell

5 Not Applicable (i.e. Retrospective case-control)

**RATE THIS SECTION**

**STRONG 1 MODERATE 2 WEAK 3	Not Applicable**

G) **INTERVENTION INTEGRITY**

**(Q1) What percentage of participants received the allocated intervention or exposure of interest?**

1 80 -100%

2 60 - 79% in taper support group 67% received all the sessions

3 less than 60%

4 Can’t tell

**(Q2) Was the consistency of the intervention measured?**

1 Yes

2 No

3 Can’t tell

**(Q3) Is it likely that subjects received an unintended intervention (contamination or co-intervention) that may influence the results?**

4 Yes

5 No

6 Can’t tell

H) **ANALYSES**

**(Q1) Indicate the unit of allocation (circle one)**

community

organization/institution

practice/office

individual

**(Q2) Indicate the unit of analysis (circle one)**

community

organization/institution

practice/office

individual

**(Q3) Are the statistical methods appropriate for the study design?**

1 Yes

2 No

3 Can’t tell

**(Q4) Is the analysis performed by intervention allocation status (i.e. intention to treat) rather than the actual intervention received?**

1 Yes

2 No

3 Can’t tell

**GLOBAL RATING**

**COMPONENT RATINGS**

Please transcribe the information from the grey boxes on pages 1-4 onto this page. See the dictionary on how to rate this section.

| A  Selection Bias | STRONG  1 | MODERATE  2 | WEAK  3 |  |
| --- | --- | --- | --- | --- |
| B  Study Design | **STRONG**  **1** | **MODERATE**  **2** | **WEAK**  **3** |  |
| C  Confounders | **STRONG**  **1** | **MODERATE**  **2** | **WEAK**  **3** |  |
| D  Blinding | **STRONG**  **1** | **MODERATE**  **2** | **WEAK**  **3** |  |
| D  Data Collecting Method | **STRONG**  **1** | **MODERATE**  **2** | **WEAK**  **3** |  |
| F  Withdrawals and Dropouts | **STRONG**  **1** | **MODERATE**  **2** | **WEAK**  **3** | Not applicable |

**GLOBAL RATING FOR THIS PAPER** (circle one):

1 STRONG (no WEAK ratings)

2 MODERATE (one WEAK rating)

3 WEAK (two or more WEAK ratings)

With both reviewers discussing the ratings:

Is there a discrepancy between the two reviewers with component (A-F) ratings?

Yes

No

If yes, Indicate the reason for the discrepancy

1 Oversight

2 Differences in interpretation of criteria

3 Differences in interpretation of study

Final Decision of both reviewers:

**1 STRONG**

**2 MODERATE
3 WEAK**

Studies Quality - Risk of Bias

**The Effective Public Health Practice Project (EPHPP) checklist criteria for each study**

# **STUDY: Vogler et al., 2017**

**COMPONENT RATINGS**

A) **SELECTION OF BIAS**

**(Q1) Are the individuals selected to participate in the study likely to be representative of the target population?**

1 Very likely

2 Somewhat likely

3 Not likely clinic referrals, self-referrals and monetary compensation for attending

4 Can’t tell

**(Q2) What percentage of selected individuals agreed to participate?**

1 80 - 100% agreement

2 60 – 79% agreement

3 less than 60% agreement

4 Not applicable

5 Can’t tell

**RATE THIS SECTION**

**STRONG 1**

**MODERATE 2**

**WEAK 3**

B) **STUDY DESIGN**

Indicate the study design

1 Randomized controlled trial

2 Controlled clinical trial

3 Cohort analytic (two group pre + post)

4 Case-control

5 Cohort (one group pre + post (before and after)

6 Interrupted time series

7 Other specify ____________________________

8 Can’t tell

Was the study described as randomized? If NO, go to Component C.

No Yes

If Yes, was the method of randomization described? (See dictionary)

No Yes

If Yes, was the method appropriate?

**RATE THIS SECTION**

**STRONG 1 MODERATE 2 WEAK 3**

C) **CONFOUNDERS**

**(Q1) Were there important differences between groups prior to the intervention?**

1 Yes

2 No

3 Can’t tell

The following are examples of confounders:

1 Race

2 Sex

3 Marital status/family

4 Age

5 SES (income or class)

6 Education

7 Health status

8 Pre-intervention score on outcome measure

(**Q2) If yes, indicate the percentage of relevant confounders that were controlled (either in the design (e.g. stratification, matching) or analysis)?**

1 80 – 100% (most)

2 60 – 79% (some)

3 Less than 60% (few or none)

4 Can’t Tell

**RATE THIS SECTION**

**STRONG 1 MODERATE 2 WEAK 3**

D) **BLINDING**

**(Q1) Was (were) the outcome assessor(s) aware of the intervention or exposure status of participants?**

1 Yes

2 No

3 Can’t tell no information provided

**(Q2) Were the study participants aware of the research question?**

1 Yes

2 No

3 Can’t tell

**RATE THIS SECTION**

**STRONG 1 MODERATE 2 WEAK 3**

E) **DATA COLLECTION METHODS**

**(Q1) Were data collection tools shown to be valid?**

1 Yes self reported and health records

2 No

3 Can’t tell

**(Q2) Were data collection tools shown to be reliable?**

1 Yes

2 No

3 Can’t tell

**RATE THIS SECTION**

**STRONG 1 MODERATE 2 WEAK 3**

F) **WITHDRAWALS AND DROP-OUTS**

**(Q1) Were withdrawals and drop-outs reported in terms of numbers and/or reasons per group?**

1 Yes

2 No

3 Can’t tell

4 Not Applicable (i.e. one time surveys or interviews)

**(Q2) Indicate the percentage of participants completing the study. (If the percentage differs by groups, record the lowest).**

1 80 -100%

2 60 - 79%

3 less than 60%

4 Can’t tell not reported

5 Not Applicable (i.e. Retrospective case-control)

**RATE THIS SECTION**

**STRONG 1 MODERATE 2 WEAK 3	Not Applicable**

G) **INTERVENTION INTEGRITY**

**(Q1) What percentage of participants received the allocated intervention or exposure of interest?**

1 80 -100%

2 60 - 79%

3 less than 60%

4 Can’t tell

**(Q2) Was the consistency of the intervention measured?**

1 Yes

2 No

3 Can’t tell

**(Q3) Is it likely that subjects received an unintended intervention (contamination or co-intervention) that may influence the results?**

4 Yes

5 No

6 Can’t tell

H) **ANALYSES**

**(Q1) Indicate the unit of allocation (circle one)**

community

organization/institution

practice/office

individual

**(Q2) Indicate the unit of analysis (circle one)**

community

organization/institution

practice/office

individual

**(Q3) Are the statistical methods appropriate for the study design?**

1 Yes

2 No

3 Can’t tell

**(Q4) Is the analysis performed by intervention allocation status (i.e. intention to treat) rather than the actual intervention received?**

1 Yes

2 No

3 Can’t tell

**GLOBAL RATING**

**COMPONENT RATINGS**

Please transcribe the information from the gray boxes on pages 1-4 onto this page. See dictionary on how to rate this section.

| A  Selection Bias | STRONG  1 | MODERATE  2 | WEAK  3 |  |
| --- | --- | --- | --- | --- |
| B  Study Design | **STRONG**  **1** | **MODERATE**  **2** | **WEAK**  **3** |  |
| C  Confounders | **STRONG**  **1** | **MODERATE**  **2** | **WEAK**  **3** |  |
| D  Blinding | **STRONG**  **1** | **MODERATE**  **2** | **WEAK**  **3** |  |
| D  Data Collecting Method | **STRONG**  **1** | **MODERATE**  **2** | **WEAK**  **3** |  |
| F  Withdrawals and Dropouts | **STRONG**  **1** | **MODERATE**  **2** | **WEAK**  **3** | Not applicable |

**GLOBAL RATING FOR THIS PAPER** (circle one):

1 STRONG (no WEAK ratings)

2 MODERATE (one WEAK rating)

3 WEAK (two or more WEAK ratings)

With both reviewers discussing the ratings:

Is there a discrepancy between the two reviewers with component (A-F) ratings?

Yes

No

If yes, Indicate the reason for the discrepancy

1 Oversight

2 Differences in interpretation of criteria

3 Differences in interpretation of study

Final Decision of both reviewers:

**1 STRONG**

**2 MODERATE
3 WEAK**

Studies Quality - Risk of Bias

**The Effective Public Health Practice Project (EPHPP) checklist criteria for each study**

# STUDY: Wilson et al., 2023

**COMPONENT RATINGS**

A) **SELECTION OF BIAS**

**(Q1) Are the individuals selected to participate in the study likely to be representative of the target population?**

1 Very likely

2 Somewhat likely	clinical registration

3 Not likely

4 Can’t tell

**(Q2) What percentage of selected individuals agreed to participate?**

1 80 - 100% agreement

2 60 – 79% agreement from 589 eligible, 402 were randomised

3 less than 60% agreement

4 Not applicable

5 Can’t tell

**RATE THIS SECTION**

**STRONG 1**

**MODERATE 2**

**WEAK 3**

B) **STUDY DESIGN**

Indicate the study design

1 Randomized controlled trial

2 Controlled clinical trial

3 Cohort analytic (two group pre + post)

4 Case-control

5 Cohort (one group pre + post (before and after))

6 Interrupted time series

7 Other specify ____________________________

8 Can’t tell

Was the study described as randomized? If NO, go to Component C.

No Yes

If Yes, was the method of randomization described? (See dictionary)

No Yes

If Yes, was the method appropriate? Yes. ‘ Research Electronic Data Capture performed the randomization process with the randomization sequence unknown to research staff. ‘

**RATE THIS SECTION**

**STRONG 1 MODERATE 2 WEAK 3**

C) **CONFOUNDERS**

**(Q1) Were there important differences between groups prior to the intervention?**

1 Yes

2 No baseline characteristics did not differ significantly between groups

3 Can’t tell

The following are examples of confounders:

1 Race

2 Sex

3 Marital status/family

4 Age

5 SES (income or class)

6 Education

7 Health status

8 Pre-intervention score on outcome measure

(**Q2) If yes, indicate the percentage of relevant confounders that were controlled (either in the design (e.g. stratification, matching) or analysis)?**

1 80 – 100% (most)

2 60 – 79% (some)

3 Less than 60% (few or none)

4 Can’t Tell

**RATE THIS SECTION**

**STRONG 1 MODERATE 2 WEAK 3**

D) **BLINDING**

**(Q1) Was (were) the outcome assessor(s) aware of the intervention or exposure status of participants?**

1 Yes

2 No

3 Can’t tell

**(Q2) Were the study participants aware of the research question?**

1 Yes

2 No

3 Can’t tell

**RATE THIS SECTION**

**STRONG 1 MODERATE 2 WEAK 3**

E) **DATA COLLECTION METHODS**

**(Q1) Were data collection tools shown to be valid?**

1 Yes

2 No

3 Can’t tell

**(Q2) Were data collection tools shown to be reliable?**

1 Yes

2 No

3 Can’t tell

**RATE THIS SECTION**

**STRONG 1 MODERATE 2 WEAK 3**

F) **WITHDRAWALS AND DROP-OUTS**

**(Q1) Were withdrawals and drop-outs reported in terms of numbers and/or reasons per group?**

1 Yes

2 No

3 Can’t tell

4 Not Applicable (i.e. one time surveys or interviews)

**(Q2) Indicate the percentage of participants completing the study. (If the percentage differs by groups, record the lowest).**

1 80 -100%

2 60 - 79%

3 less than 60%

4 Can’t tell

5 Not Applicable (i.e. Retrospective case-control)

**RATE THIS SECTION**

**STRONG 1 MODERATE 2 WEAK 3	Not Applicable**

G) **INTERVENTION INTEGRITY**

**(Q1) What percentage of participants received the allocated intervention or exposure of interest?**

1 80 -100%

2 60 - 79%

3 less than 60%

4 Can’t tell

**(Q2) Was the consistency of the intervention measured?**

1 Yes

2 No

3 Can’t tell

**(Q3) Is it likely that subjects received an unintended intervention (contamination or co-intervention) that may influence the results?**

4 Yes

5 No

6 Can’t tell

H) **ANALYSES**

**(Q1) Indicate the unit of allocation (circle one)**

community

organization/institution

practice/office

individual

**(Q2) Indicate the unit of analysis (circle one)**

community

organization/institution

practice/office

individual

**(Q3) Are the statistical methods appropriate for the study design?**

1 Yes

2 No

3 Can’t tell

**(Q4) Is the analysis performed by intervention allocation status (i.e. intention to treat) rather than the actual intervention received?**

1 Yes

2 No

3 Can’t tell

**GLOBAL RATING**

**COMPONENT RATINGS**

Please transcribe the information from the gray boxes on pages 1-4 onto this page. See dictionary on how to rate this section.

| A  Selection Bias | STRONG  1 | MODERATE  2 | WEAK  3 |  |
| --- | --- | --- | --- | --- |
| B  Study Design | **STRONG**  **1** | **MODERATE**  **2** | **WEAK**  **3** |  |
| C  Confounders | **STRONG**  **1** | **MODERATE**  **2** | **WEAK**  **3** |  |
| D  Blinding | **STRONG**  **1** | **MODERATE**  **2** | **WEAK**  **3** |  |
| D  Data Collecting Method | **STRONG**  **1** | **MODERATE**  **2** | **WEAK**  **3** |  |
| F  Withdrawals and Dropouts | **STRONG**  **1** | **MODERATE**  **2** | **WEAK**  **3** | Not applicable |

**GLOBAL RATING FOR THIS PAPER** (circle one):

1 STRONG (no WEAK ratings)

2 MODERATE (one WEAK rating)

3 WEAK (two or more WEAK ratings)

With both reviewers discussing the ratings:

Is there a discrepancy between the two reviewers with component (A-F) ratings?

Yes

No

If yes, Indicate the reason for the discrepancy

1 Oversight

2 Differences in interpretation of criteria

3 Differences in interpretation of study

Final Decision of both reviewers:

**1 STRONG**

**2 MODERATE
3 WEAK**
