# Supplementary material for: Behaviour change interventions to reduce opioids in chronic non-cancer pain in primary care: A systematic review and mapping of behavior change techniques
Source: Medicine (Baltimore). 2026 Aug 7;105(32):e50106. doi: 10.1097/MD.0000000000050106 (PMC13456882; doi:10.1097/MD.0000000000050106)
Supplement: Supplementary file 5 [file medi-105-e50106-s005.docx]

S3 Table. BCTs, intervention techniques linked to behaviour change Techniques Taxonomy version 1

|  | Bushey et al., 2022 | Vogler et al., 2017 | Mehl-Madrona et al., 2016 | Scott et al, 2020 | Seal et al, 2020 | Sullivan et al., 2017 | DeBar et al., 2022 | Garland et al., 2020 | Garland et al., 2022 | Sandhu et al., 2023 | Wilson et al., 2023 | Borsari et al., 2021 | Darnall et al., 2018 |
| --- | --- | --- | --- | --- | --- | --- | --- | --- | --- | --- | --- | --- | --- |
| *Goals and Planning* |  |  |  |  |  |  |  |  |  |  |  |  |  |
| 1.1. Goal Setting (behaviour) | X+ | x+ |  | x++ |  | x++ | x++ |  |  | x++ |  | x++ | x+ |
| 1.2. Problem Solving |  |  |  |  |  | x++ | x++ |  |  | x++ |  | x++ |  |
| 1.3. Goal setting (outcome) |  | x++ | x++ | x++ | x++ | x++ | X++ |  |  | x++ | x++ | x++ | x++ |
| 1.4. Action Planning | X++ | x + |  | x++ | x+ | x++ | x++ |  |  | x++ | x++ | x++ |  |
| 1.5 Review Behaviour Goal(s) | X++ |  |  |  |  |  | x++ |  |  |  | x++ | x++ |  |
| 1.6 Discrepancy between current behaviour and goal |  |  |  |  |  | x++ |  |  |  |  |  | x++ |  |
| 1.7 Review outcome goal(s) | X++ |  |  |  |  | x++ | x++ |  |  | x++ | x++ |  | x++ |
| 1.8 Behavioural Contract | X++ |  | x++ | x++ |  | x++ |  |  |  | x++ |  | x++ | x++ |
| 1.9 Commitment |  |  |  |  |  | x++ |  |  |  | x++ |  |  |  |
| *Feedback and Monitoring* |  |  |  |  |  |  |  |  |  |  |  |  |  |
| 2.1 Monitoring of behaviour by other without feedback |  |  |  |  |  |  |  |  |  |  |  |  |  |
| 2.2 Feedback on behaviour |  |  |  |  |  |  | x++ |  |  |  |  |  | x+ |
| 2.3 Self-monitoring of behaviour |  |  |  |  |  |  |  | x++ | x++ |  | x++ |  |  |
| 2.4 Self-monitoring of outcome(s) of behaviour |  |  |  |  |  |  |  |  |  |  | x++ |  |  |
| 2.5 Monitoring of outcome(s) of behaviour without feedback |  |  |  |  |  |  |  |  |  |  |  |  |  |
| 2.6 Biofeedback |  |  |  |  |  |  |  |  |  |  |  |  |  |
| 2.7 Feedback on outcome(s) of behaviour |  |  |  |  |  | x++ | x++ |  |  |  |  | x++ |  |
| *Social Support* |  |  |  |  |  |  |  |  |  |  |  |  |  |
| 3.1 Social Support (unspecified) |  |  |  |  |  |  |  |  |  |  |  |  |  |
| 3.2 Social support (Pratical) |  |  |  |  |  |  |  |  |  |  |  |  |  |
| 3.3 Social support (emotional) |  |  |  |  |  |  |  |  |  | x++ |  |  |  |
| *Shaping Knowledge* |  |  |  |  |  |  |  |  |  |  |  |  |  |
| 4.1 Instruction on how to perform the behaviour |  | x++ | x++ | x++ |  | x++ | x++ | x++ | x++ | x++ | x++ |  | x+ |
| 4.2 Information about antecedents |  | x++ | x+ |  |  | x+ | x++ | x++ | x++ | x++ | x++ |  |  |
| 4.3 Re-attribution |  | x++ | x++ |  |  | x++ | x++ | x++ | x+ | x++ | x++ |  |  |
| 4.4 Behavioural experiments |  |  |  |  |  |  |  |  |  |  |  |  |  |
| *Natural Consequences* |  |  |  |  |  |  |  |  |  |  |  |  |  |
| 5.1 Information about health consequences | x++ | x++ | x++ | x++ |  | x++ | x+ | x++ | x++ | x++ | x++ |  | x++ |
| 5.2 Salience of consequences |  |  |  |  |  |  |  |  |  |  |  |  |  |
| 5.3 Information about social and environmental consequences |  |  |  |  |  |  |  | x+ | x+ |  |  |  |  |
| 5.4 Monitoring of emotional Consequences |  |  |  |  |  |  | x+ |  |  |  |  |  |  |
| 5.5 Antecipated regret |  |  |  |  |  |  | x++ | x+ | x+ | x++ |  |  |  |
| 5.6 Information about emotional consequences |  |  | x+ | x++ |  | x++ | x++ | x+ | x+ |  | x++ |  |  |
| Comparison of behaviour |  |  |  |  |  |  |  |  |  |  |  |  |  |
| 6.1 Demonstration of the behaviour |  | x + | x++ | x++ |  | x++ | x++ |  |  | x++ |  |  |  |
| 6.2 Social comparison |  | x+ | x+ |  |  | x++ |  | x+ | x+ | x++ |  |  |  |
| 6.3 Information about others' approval |  |  |  |  |  |  |  |  |  |  |  |  |  |
| *Associations* |  |  |  |  |  |  |  |  |  |  |  |  |  |
| 7.1 Prompts/cues |  |  |  |  |  |  |  |  |  |  |  |  |  |
| 7.2 Cue signalling reward |  |  |  |  |  |  |  |  |  |  |  |  |  |
| 7.3 Reduce prompts/cues |  |  |  |  |  |  |  |  |  |  |  |  |  |
| 7.4 Remove access to the reward |  |  | x+ |  |  |  |  |  |  |  |  |  |  |
| 7.5 Remove aversive stimulus |  |  |  |  |  |  |  |  |  | x+ |  |  |  |
| 7.6 Satiation |  |  |  |  |  |  |  |  |  |  |  |  |  |
| 7.7 Exposure |  |  |  |  |  |  |  |  |  |  |  |  |  |
| 7.8 Associative learning |  |  |  |  |  |  |  |  |  |  |  |  |  |
| *Repetition and substitution* |  |  |  |  |  |  |  |  |  |  |  |  |  |
| 8.1 Behavioural practice/ rehearsal |  |  |  | x+ |  | x+ | x++ | x++ | x++ | x++ | X+ |  |  |
| 8.2 Behaviour substitution |  |  |  | x+ | x+ |  | x++ | x++ | x++ | x++ | x++ |  |  |
| 8.3 Habit formation |  |  |  |  |  |  | x++ | x++ | x++ |  | X+ |  |  |
| 8.4 habit reversal |  |  |  |  |  |  | x++ | x++ | x++ |  |  |  |  |
| 8.5 Overcorrection |  |  |  |  |  |  |  |  |  |  |  |  |  |
| 8.6 Generalisation of target behaviour |  |  |  |  |  |  |  |  |  |  |  |  |  |
| 8.7 Graded tasks |  |  |  | x++ | x+ | x++ | x++ |  |  | x++ | x++ |  | x+? |
| *Comparison of Outcomes* |  |  |  |  |  |  |  |  |  |  |  |  |  |
| 9.1 Credible Source | X++ | x++ | x++ | x++ | x++ | x++ | x++ | x++ | x++ | x++ |  | x++ |  |
| 9.2 Pros and Cons |  |  |  |  |  |  |  |  |  | x++ |  |  |  |
| 9.3 Comparative imagining of future outcomes |  |  |  |  |  |  |  |  |  | x++ |  |  |  |
| *Reward and threat* |  |  |  |  |  |  |  |  |  |  |  |  |  |
| 10.1 material incentive (behaviour) |  |  |  |  |  |  |  |  |  |  |  |  |  |
| 10.2 Material reward (behaviour) |  |  | x++ |  |  |  |  |  |  |  |  |  |  |
| 10.3 Non-specific reward |  |  |  |  |  |  |  |  |  |  | x++ |  |  |
| 10.4 Social reward |  |  |  |  |  |  |  |  |  | x++ | x++ |  |  |
| 10.5 Social incentive |  |  |  |  |  |  |  |  |  |  |  |  |  |
| 10.6 Non- specific incentive |  |  |  |  |  |  |  |  |  |  |  |  |  |
| 10.7 Self-incentive |  |  |  |  |  |  |  |  |  |  | x++ |  |  |
| 10.8 Incentive (outcome) |  |  |  |  |  |  |  |  |  |  |  |  |  |
| 10.9 Self-reward |  |  |  |  |  |  |  |  |  |  | x++ |  |  |
| 10.10 Reward (outcome) |  |  |  |  |  |  | x+ |  |  |  | x++ |  |  |
| 10.11 Future Punishment |  |  | x++ |  |  |  |  |  |  |  |  |  |  |
| *Regulation* |  |  |  |  |  |  |  |  |  |  |  |  |  |
| 11.1 Pharmacological support | X++ |  |  |  |  |  |  |  |  |  |  |  |  |
| 11.2 Reduce negative emotions |  |  | x++ | x++ | x+ | x++ | x++ | x++ | x++ | x++ | x++ |  |  |
| 11.3 Conserving Mental Resources |  |  |  |  |  |  |  |  |  |  |  |  |  |
| 11.4 Paradoxical instructions |  |  |  |  |  |  |  |  |  |  |  |  |  |
| *Antecedents* |  |  |  |  |  |  |  |  |  |  |  |  |  |
| 12.1 Restructuring the physical environment |  |  |  |  |  |  |  |  |  | x+ |  |  |  |
| 12.2 Restructuring the social environment |  |  |  |  |  |  |  |  |  |  |  |  |  |
| 12.3 avoidance/ reducing exposure to cues for the behaviour |  |  |  |  |  |  |  |  |  | x+ |  |  |  |
| 12.4 Distraction |  |  | x++ | x++ | x++ | x++ | x++ | x++ | x++ | x++ | x++ |  |  |
| 12.5 Adding objects to the environment |  |  |  |  |  |  |  |  |  |  |  |  |  |
| 12.6 Body changes |  |  | x++ | x++ | x++ | x++ | x++ | x++ | x++ | x++ | x++ |  |  |
| *Identity* |  |  |  |  |  |  |  |  |  |  |  |  |  |
| 13.1 Identification of self as a role model |  |  |  |  |  |  |  |  |  | x++ |  |  |  |
| 13.2 Framing/ reframing |  |  | x+ | x++ | x++ | x++ | x++ | x++ | x++ | x++ | x+? |  |  |
| 13.3 Incompatible beliefs |  |  |  |  |  |  |  |  |  | x+ |  |  |  |
| 13.4 Valued self-identify |  |  |  | x++ |  |  |  |  |  |  |  |  |  |
| 13.5 Identity associated with changed behaviour |  |  |  |  |  |  |  |  |  | x+ |  |  |  |
| *Scheduled consequences* |  |  |  |  |  |  |  |  |  |  |  |  |  |
| 14.1 Behaviour cost |  |  | x++ |  |  |  |  |  |  |  |  |  |  |
| 14.2 Punishment |  |  | x+ |  |  |  |  |  |  |  |  |  |  |
| 14.3 Remove reward |  |  |  |  |  |  |  |  |  |  |  |  |  |
| 14.4 Reward approximation |  |  |  |  |  |  | x+ |  |  |  |  |  |  |
| 14.5 Rewarding completion |  |  |  |  |  |  |  |  |  |  |  |  |  |
| 14.6 Situation- specific reward |  |  |  |  |  |  |  |  |  |  |  |  |  |
| 14.7 Reward incompatible behaviour |  |  |  |  |  |  |  |  |  |  |  |  |  |
| 14.8 Reward alternative behaviour |  |  |  |  |  |  |  |  |  |  |  |  |  |
| 14.9 reduce reward frequency |  |  |  |  |  |  |  |  |  |  |  |  |  |
| 14.10 Remove punishment |  |  |  |  |  |  |  |  |  |  |  |  |  |
| *Self-belief* |  |  |  |  |  |  |  |  |  |  |  |  |  |
| 15.1 Verbal persuasion about capability |  |  |  | x++ |  | x++ |  |  |  | x++ |  | x+ |  |
| 15.2 Mental rehearsal of successful performance |  |  |  |  |  | x+ |  |  |  |  |  |  |  |
| 15.3 Focus on past success |  |  |  | x+ |  |  |  |  |  | x++ |  | x+ |  |
| 15.4 Self-talk |  |  |  |  |  | x+ |  |  |  |  |  | X+ |  |
| *Covert learning* |  |  |  |  |  |  |  |  |  |  |  |  |  |
| 16.1 Imaginary punishment |  |  |  |  |  |  |  |  |  |  |  |  |  |
| 16.2 Imaginary reward |  |  |  |  |  |  |  |  |  |  |  |  |  |
| 16.3 Vicarious consequences |  |  |  |  |  |  |  |  |  |  |  |  |  |
