# Supplementary material for: Behaviour change interventions to reduce opioids in chronic non-cancer pain in primary care: A systematic review and mapping of behavior change techniques
Source: Medicine (Baltimore). 2026 Aug 7;105(32):e50106. doi: 10.1097/MD.0000000000050106 (PMC13456882; doi:10.1097/MD.0000000000050106)
Supplement: Supplementary file 6 [file medi-105-e50106-s006.docx]

S4 Table. BCTs, intervention techniques linked with opioid Outcome

|  | Mehl-Madrona et al., 2016 | Scott et al, 2020 | Seal et al, 2020 | Garland et all., 2020 | Garland et al., 2022 | Sandhu et al., 2023 | Wilson et al., 2023 | Darnall et al., 2018 | TOTAL |
| --- | --- | --- | --- | --- | --- | --- | --- | --- | --- |
| *Goals and Planning* |  |  |  |  |  |  |  |  |  |
| 1.1. Goal Setting (behaviour) |  | x++ |  |  |  | x++ |  | x+? | 3 |
| 1.2. Problem Solving |  |  |  |  |  | x++ |  |  | 1 |
| 1.3. Goal setting (outcome) | x++ | x++ | x++ |  |  | x++ | x++ | x++ | 6 |
| 1.4. Action Planning |  | x++ | x+ |  |  | x++ | x++ |  | 4 |
| 1.5 Review Behaviour Goal(s) |  |  |  |  |  |  | x++ |  | 1 |
| 1.7 Review outcome goal(s) |  |  |  |  |  | x++ | x++ | x++ | 3 |
| 1.8 Behavioural Contract | x++ | x++ |  |  |  | x++ |  | x++ | 4 |
| 1.9 Commitment |  |  |  |  |  | x++ |  |  | 1 |
| *Feedback and Monitoring* |  |  |  |  |  |  |  |  |  |
| 2.2 Feedback on behaviour |  |  |  |  |  |  |  | x+ | 1 |
| 2.3 Self-monitoring of behaviour |  |  |  | x++ | x++ |  | x++ |  | 3 |
| 2.4 Self-monitoring of outcome(s) of behaviour |  |  |  |  |  |  | x++ |  | 1 |
| *Social Support* |  |  |  |  |  |  |  |  |  |
| 3.3 Social support (emotional) |  |  |  |  |  | x++ |  |  | 1 |
| *Shaping Knowledge* |  |  |  |  |  |  |  |  |  |
| 4.1 Instruction on how to perform the behaviour | x++ | x++ |  | x++ | x++ | x++ | x++ | x+ | 7 |
| 4.2 Information about antecedents | x+ |  |  | x++ | x++ | x++ | x++ |  | 5 |
| 4.3 Re-attribuition | x++ |  |  | x++ | x+ | x++ | x++ |  | 5 |
| *Natural Consequences* |  |  |  |  |  |  |  |  |  |
| 5.1 Information about health consequences | x++ | x++ |  | x++ | x++ | x++ | x++ | x++ | 7 |
| 5.3 Information about social and environmental consequences |  |  |  | x+ | x+ |  |  |  | 2 |
| 5.5 Antecipated regret |  |  |  | x+ | x+ | x++ |  |  | 3 |
| 5.6 Information about emotional consequences | x+ | x++ |  | x+ | x+ |  | x++ |  | 5 |
| Comparison of behaviour |  |  |  |  |  |  |  |  |  |
| 6.1 Demonstration of the behaviour | x++ | x++ |  |  |  | x++ |  |  | 3 |
| 6.2 Social comparison | x+ |  |  | x+ | x+ | x++ |  |  | 4 |
| *Associations* |  |  |  |  |  |  |  |  |  |
| 7.4 Remove access to the reward | x+ |  |  |  |  |  |  |  | 1 |
| 7.5 Remove aversive stimulus |  |  |  |  |  | x+? |  |  | 1 |
| *Repetition and substitution* |  |  |  |  |  |  |  |  |  |
| 8.1 Behavioural practice/ rehearsal |  | x+ |  | x++ | x++ | x++ | X+ |  | 5 |
| 8.2 Behaviour substitution |  | x+ | x+ | x++ | x++ | x++ | x++ |  | 6 |
| 8.3 Habit formation |  |  |  | x++ | x++ |  | X+ |  | 3 |
| 8.4 Habit reversal |  |  |  | x++ | x++ |  |  |  | 2 |
| 8.7 Graded tasks |  | x++ | x+ |  |  | x++ | x++ | x+ | 5 |
| *Comparison of Outcomes* |  |  |  |  |  |  |  |  |  |
| 9.1 Credible Source | x++ | x++ | x++ | x++ | x++ | x++ |  |  | 6 |
| 9.2 Pros and Cons |  |  |  |  |  | x++ |  |  | 1 |
| 9.3 Comparative imagining of future outcomes |  |  |  |  |  | x++ |  |  | 1 |
| *Reward and threat* |  |  |  |  |  |  |  |  |  |
| 10.2 Material reward (behaviour) | x++ |  |  |  |  |  |  |  | 1 |
| 10.3 Non-specific reward |  |  |  |  |  |  | x++ |  | 1 |
| 10.4 Social reward |  |  |  |  |  | x++ | x++ |  | 2 |
| 10.7 Self-incentive |  |  |  |  |  |  | x++ |  | 1 |
| 10.9 Self-reward |  |  |  |  |  |  | x++ |  | 1 |
| 10.10 Reward (outcome) |  |  |  |  |  |  | x++ |  | 1 |
| 10.11 Future Punishment | x++ |  |  |  |  |  |  |  | 1 |
| *Regulation* |  |  |  |  |  |  |  |  |  |
| 11.2 Reduce negative emotions | x++ | x++ | x+ | x++ | x++ | x++ | x++ |  | 7 |
| *Antecedents* |  |  |  |  |  |  |  |  |  |
| 12.1 Restructuring the physical environment |  |  |  |  |  | x+ |  |  | 1 |
| 12.3 Avoidance/ reducing exposure to cues for the behaviour |  |  |  |  |  | x+ |  |  | 1 |
| 12.4 Distraction | x++ | x++ | x++ | x++ | x++ | x++ | x++ |  | 7 |
| 12.6 Body changes | x++ | x++ | x++ | x++ | x++ | x++ | x++ |  | 7 |
| *Identity* |  |  |  |  |  |  |  |  |  |
| 13.2 Framing/ reframing | x+ | x++ | x++ | x++ | x++ | x++ | x+ |  | 7 |
| 13.3 Incompatible beliefs |  |  |  |  |  | x+ |  |  | 1 |
| 13.4 Valued self-identify |  | x++ |  |  |  |  |  |  | 1 |
| 13.5 Identity associated with changed behaviour |  |  |  |  |  | x+ |  |  | 1 |
| *Scheduled consequences* |  |  |  |  |  |  |  |  |  |
| 14.1 Behaviour cost | x++ |  |  |  |  |  |  |  | 1 |
| 14.2 Punishment | x+ |  |  |  |  |  |  |  | 1 |
| *Self-belief* |  |  |  |  |  |  |  |  |  |
| 15.1 Verbal persuasion about capability |  | x++ |  |  |  | x++ |  |  | 2 |
| 15.3 Focus on past success |  | x+ |  |  |  | x++ |  |  | 2 |
| TOTAL | 20 | 19 | 9 | 18 | 18 | 34 | 24 | 8 |  |
