# Supplementary material for: Behaviour change interventions to reduce opioids in chronic non-cancer pain in primary care: A systematic review and mapping of behavior change techniques
Source: Medicine (Baltimore). 2026 Aug 7;105(32):e50106. doi: 10.1097/MD.0000000000050106 (PMC13456882; doi:10.1097/MD.0000000000050106)
Supplement: Supplementary file 7 [file medi-105-e50106-s007.docx]

S5 Table. BCTs, intervention techniques linked to Opioid Misuse Risk Outcomes

|  | Garland et all., 2020 | Garland et al., 2022 |
| --- | --- | --- |
|  |  |  |
| *Feedback and Monitoring* |  |  |
| 2.3 Self-monitoring of behaviour | x++ | x++ |
| *Shaping Knowledge* |  |  |
| 4.1 Instruction on how to perform the behaviour | x++ | x++ |
| 4.2 Information about antecedents | x++ | x++ |
| 4.3 Re-attribuition | x++ | x+ |
| *Natural Consequences* |  |  |
| 5.1 Information about health consequences | x++ | x++ |
| 5.3 Information about social and environmental consequences | x+ | x+ |
| 5.5 Antecipated regret | x+ | x+ |
| 5.6 Information about emotional consequences | x+ | x+ |
| Comparison of behaviour |  |  |
| 6.2 Social comparison | x+ | x+ |
| *Repetition and substitution* |  |  |
| 8.1 Behavioural practice/ rehearsal | x++ | x++ |
| 8.2 behaviour substitution | x++ | x++ |
| 8.3 Habit formation | x++ | x++ |
| 8.4 habit reversal | x++ | x++ |
| *Comparison of Outcomes* |  |  |
| 9.1 Credible Source | x++ | x++ |
| *Regulation* |  |  |
| 11.2 Reduce negative emotions | x++ | x++ |
| *Antecedents* |  |  |
| 12.4 Distraction | x++ | x++ |
| 12.6 Body changes | x++ | x++ |
| *Identity* |  |  |
| 13.2 Framing/ reframing | x++ | x++ |
