# Supplementary material for: Behaviour change interventions to reduce opioids in chronic non-cancer pain in primary care: A systematic review and mapping of behavior change techniques
Source: Medicine (Baltimore). 2026 Aug 7;105(32):e50106. doi: 10.1097/MD.0000000000050106 (PMC13456882; doi:10.1097/MD.0000000000050106)
Supplement: Supplementary file 8 [file medi-105-e50106-s008.docx]

S6 Table. BCTs, intervention techniques linked Pain Impact Outcomes

**Behaviour change interventions to reduce opioids in chronic non-cancer pain in primary care: a systematic review and mapping of behaviour change techniques**

Andreia Ramos Silva, Catharine Montgomery, Bernhard Frank, Katie Herron, Helen Poole

|  | Bushey et al., 2022 | Mehl-Madrona et al., 2016 | Sullivan et al., 2017 | DeBar et al., 2022 | Garland et all., 2020 | Garland et al., 2022 |
| --- | --- | --- | --- | --- | --- | --- |
| *Goals and Planning* |  |  |  |  |  |  |
| 1.1. Goal Setting (behaviour) | X+ |  | x++ | x++ |  |  |
| 1.2. Problem Solving |  |  | x++ | x++ |  |  |
| 1.3. Goal setting (outcome) |  | x++ | x++ | X++ |  |  |
| 1.4. Action Planning | X++ |  | x++ | x++ |  |  |
| 1.5 Review Behaviour Goal(s) | X++ |  |  | x++ |  |  |
| 1.6 Discrepancy between current behaviour and goal |  |  | x++ |  |  |  |
| 1.7 Review outcome goal(s) | X++ |  | x++ | x++ |  |  |
| 1.8 Behavioural Contract | X++ | x++ | x++ |  |  |  |
| 1.9 Commitment |  |  | x++ |  |  |  |
| *Feedback and Monitoring* |  |  |  |  |  |  |
| 2.2 Feedback on behaviour |  |  |  | x++ |  |  |
| 2.3 Self-monitoring of behaviour |  |  |  |  | x++ | x++ |
| 2.7 Feedback on outcome(s) of behaviour |  |  | x++ | x++ |  |  |
| *Shaping Knowledge* |  |  |  |  |  |  |
| 4.1 Instruction on how to perform the behaviour |  | x++ | x++ | x++ | x++ | x++ |
| 4.2 Information about antecedents |  | x+ | x+ | x++ | x++ | x++ |
| 4.3 Re-attribution |  | x++ | x++ | x++ | x++ | x+ |
| *Natural Consequences* |  |  |  |  |  |  |
| 5.1 Information about health consequences | x++ | x++ | x++ | x+ | x++ | x++ |
| 5.3 Information about social and environmental consequences |  |  |  |  | x+ | x+ |
| 5.4 Monitoring of emotional Consequences |  |  |  | x+ |  |  |
| 5.5 Antecipated regret |  |  |  | x++ | x+ | x+ |
| 5.6 Information about emotional consequences |  | x+ | x++ | x++ | x+ | x+ |
| *Comparison of behaviour* |  |  |  |  |  |  |
| 6.1 Demonstration of the behaviour |  | x++ | x++ | x++ |  |  |
| 6.2 Social comparison |  | x+ | x++ |  | x+ | x+ |
| *Associations* |  |  |  |  |  |  |
| 7.4 Remove access to the reward |  | x+ |  |  |  |  |
| *Repetition and substitution* |  |  |  |  |  |  |
| 8.1 Behavioural practice/ rehearsal |  |  | x+ | x++ | x++ | x++ |
| 8.2 behaviour substitution |  |  |  | x++ | x++ | x++ |
| 8.3 Habit formation |  |  |  | x++ | x++ | x++ |
| 8.4 habit reversal |  |  |  | x++ | x++ | x++ |
| 8.7 Graded tasks |  |  | x++ | x++ |  |  |
| *Comparison of Outcomes* |  |  |  |  |  |  |
| 9.1 Credible Source | X++ | x++ | x++ | x++ | x++ | x++ |
| *Reward and threat* |  |  |  |  |  |  |
| 10.2 Material reward (behaviour) |  | x++ |  |  |  |  |
| 10.10 Reward (outcome) |  |  |  | x+ |  |  |
| 10.11 Future Punishment |  | x++ |  |  |  |  |
| *Regulation* |  |  |  |  |  |  |
| 11.1 Pharmacological support | X++ |  |  |  |  |  |
| 11.2 Reduce negative emotions |  | x++ | x++ | x++ | x++ | x++ |
| *Antecedents* |  |  |  |  |  |  |
| 12.4 Distraction |  | x++ | x++ | x++ | x++ | x++ |
| 12.6 Body changes |  | x++ | x++ | x++ | x++ | x++ |
| *Identity* |  |  |  |  |  |  |
| 13.2 Framing/ reframing |  | x+ | x++ | x++ | x++ | x++ |
| *Scheduled consequences* |  |  |  |  |  |  |
| 14.1 Behaviour cost |  | x++ |  |  |  |  |
| 14.2 Punishment |  | x+ |  |  |  |  |
| 14.4 Reward approximation |  |  |  | x+ |  |  |
| *Self-belief* |  |  |  |  |  |  |
| 15.1 Verbal persuasion about capability |  |  | x++ |  |  |  |
| 15.2 Mental rehearsal of successful performance |  |  | x+ |  |  |  |
| 15.4 Self-talk |  |  | x+ |  |  |  |
