# Supplementary material for: Behaviour change interventions to reduce opioids in chronic non-cancer pain in primary care: A systematic review and mapping of behavior change techniques
Source: Medicine (Baltimore). 2026 Aug 7;105(32):e50106. doi: 10.1097/MD.0000000000050106 (PMC13456882; doi:10.1097/MD.0000000000050106)
Supplement: Supplementary file 9 [file medi-105-e50106-s009.docx]

S7 Table. BCTs, intervention techniques linked to Pain Self-Efficacy Outcomes

**Behaviour change interventions to reduce opioids in chronic non-cancer pain in primary care: a systematic review and mapping of behaviour change techniques**

Andreia Ramos Silva, Catharine Montgomery, Bernhard Frank, Katie Herron, Helen Poole

|  | Sullivan et al., 2017 | Sandhu et al., 2023 | Wilson et al., 2023 |
| --- | --- | --- | --- |
| *Goals and Planning* |  |  |  |
| 1.1. Goal Setting (behaviour) | x++ | x++ |  |
| 1.2. Problem Solving | x++ | x++ |  |
| 1.3. Goal setting (outcome) | x++ | x++ | x++ |
| 1.4. Action Planning | x++ | x++ | x++ |
| 1.5 Review Behaviour Goal(s) |  |  | x++ |
| 1.6 Discrepancy between current behaviour and goal | x++ |  |  |
| 1.7 Review outcome goal(s) | x++ | x++ | x++ |
| 1.8 Behavioural Contract | x++ | x++ |  |
| 1.9 Commitment | x++ | x++ |  |
| *Feedback and Monitoring* |  |  |  |
| 2.3 Self-monitoring of behaviour |  |  | x++ |
| 2.4 Self-monitoring of outcome(s) of behaviour |  |  | x++ |
| 2.7 Feedback on outcome(s) of behaviour | x++ |  |  |
| *Social Support* |  |  |  |
| 3.3 Social support (emotional) |  | x+ |  |
| *Shaping Knowledge* |  |  |  |
| 4.1 Instruction on how to perform the behaviour | x++ | x++ | x++ |
| 4.2 Information about antecedents | x+ | x++ | x++ |
| 4.3 Re-attribuition | x++ | x++ | x++ |
| *Natural Consequences* |  |  |  |
| 5.1 Information about health consequences | x++ | x++ | x++ |
| 5.5 Antecipated regret |  | x++ |  |
| 5.6 Information about emotional consequences | x++ |  | x++ |
| *Comparison of behaviour* |  |  |  |
| 6.1 Demonstration of the behaviour | x++ | x++ |  |
| 6.2 Social comparison | x++ | x++ |  |
| *Associations* |  |  |  |
| 7.5 Remove aversive stimulus |  | x+ |  |
| *Repetition and substitution* |  |  |  |
| 8.1 Behavioural practice/ rehearsal | x+ | x++ |  |
| 8.2 behaviour substitution |  | x++ | x++ |
| 8.3 Habit formation |  |  |  |
| 8.7 Graded tasks | x++ | x++ | x++ |
| *Comparison of Outcomes* |  |  |  |
| 9.1 Credible Source | x++ | x++ |  |
| 9.2 Pros and Cons |  | x++ |  |
| 9.3 Comparative imagining of future outcomes |  | x++ |  |
| *Reward and threat* |  |  |  |
| 10.3 Non-specific reward |  |  | x++ |
| 10.4 Social reward |  | x++ | x++ |
| 10.7 Self-incentive |  |  | x++ |
| 10.9 Self-reward |  |  | x++ |
| 10.10 Reward (outcome) |  |  | x++ |
| *Regulation* |  |  |  |
| 11.2 Reduce negative emotions | x++ | x++ | x++ |
| *Antecedents* |  |  |  |
| 12.1 Restructuring the physical environment |  | x+ |  |
| 12.3 avoidance/ reducing exposure to cues for the behaviour |  | x+ |  |
| 12.4 Distraction | x++ | x++ | x++ |
| 12.6 Body changes | x++ | x++ | x++ |
| *Identity* |  |  |  |
| 13.1 Identification of self as a role model |  | x++ |  |
| 13.2 Framing/ reframing | x++ | x++ | x+ |
| 13.3 Incompatible beliefs |  | x+ |  |
| 13.5 Identity associated with changed behaviour |  | x+ |  |
| *Self-belief* |  |  |  |
| 15.1 Verbal persuasion about capability | x++ | x++ |  |
| 15.2 Mental rehearsal of successful performance | x+ |  |  |
| 15.3 Focus on past success |  | x++ |  |
| 15.4 Self-talk | x+ |  |  |
